# Phylogenetic intermixing reveals stable fly-mediated circulation of mastitis-associated bacteria in dairy settings

**DOI:** 10.1128/msystems.00215-25

**Published:** 2025-08-01

**Authors:** Andrew J. Sommer, Travis K. Worley, Panagiotis Sapountzis, Kerri L. Coon

**Affiliations:** 1 Department of Bacteriology, University of Wisconsin-Madison 5228, Madison, Wisconsin, USA; 2 Microbiology Doctoral Training Program, University of Wisconsin-Madison 5229 , Madison, Wisconsin, USA; 3 INRAE-UCA, UMR0454 MEDIS, Clermont-Ferrand, France; University of Wisconsin-Milwaukee, Milwaukee, Wisconsin, USA

**Keywords:** antimicrobial resistance, virulence, cattle, comparative genomics, microbiome, pathogen ecology, insect pests, host-microbe interactions

## Abstract

*Stomoxys* flies are common blood-feeding pests on dairy farms and are suspected carriers of pathogenic bacteria due to their close association with manure and cattle hosts. While prior studies have used amplicon sequencing and culture-dependent methodologies to characterize the composition of the *Stomoxys* microbiota, little is known about strain-level acquisition of mastitis-causing bacteria from manure by *Stomoxys* or the functional diversity of *Stomoxys*-associated taxa. In this study, we address these key knowledge gaps by using whole genome sequencing to provide the first comparative genomic analysis of *Stomoxys-*derived *Escherichia coli*, *Klebsiella pneumoniae*, and *Staphylococcaceae* isolates. Our results show that fly and manure isolates collected from the same farm system are phylogenetically interspersed, with subsequent pairwise genome alignments revealing near-identical strains and plasmids shared between the two sources. We further identify a phylogenetic clade of *Mammaliicoccus sciuri* containing known mastitis agents associated with both flies and manure. Functional analysis reveals that this clade is highly enriched in xylose metabolism genes that are rare across other *M. sciuri* lineages, suggesting potential niche differentiation within the genus. Collectively, our results provide strong evidence for the acquisition of fecal-associated bacteria by adult *Stomoxys* flies, confirming the link between biting muscid flies and manure habitats. The intermixing of fly and manure isolates in clinically relevant taxonomic groups strongly suggests that flies serve as carriers of opportunistic mastitis-causing or other fecal-borne pathogens and may serve as important vehicles of pathogen dissemination across the dairy farm environment.

**IMPORTANCE:**

Bovine mastitis causes up to $32 billion dollars in losses annually in the global dairy industry. Opportunistic intramammary pathogens can be transmitted through incidental contact with bacteria in environmental reservoirs like manure. However, factors affecting the abundance, persistence, and spread of these bacteria are not well understood. Our research shows that mastitis pathogens are present in the guts of blood-feeding *Stomoxys* (stable) flies, which develop in cow feces and bite cows. Genomic analysis of isolates from flies, manure, and mastitis cases reveals that strains and antimicrobial resistance genes are shared between these sources. Further analysis of fly gut isolates shows virulence factors and possible niche specialization, identifying fly-associated clades with known mastitis agents from mastitic cows. This strongly suggests that *Stomoxys* flies play a role in the carriage and circulation of bovine mastitis pathogens from manure in dairy settings.

## INTRODUCTION

The effective control of infectious diseases across agricultural systems is crucial for ensuring the economic stability of the global food system. On commercial dairy farms, strict hygiene practices and bio-control programs are often implemented to limit the spread of animal-borne pathogens to farm workers, other livestock, or through the food system to consumers (1–3). While much attention has been given to the surveillance of pathogens in cattle or food products (4–6), far less is known about the role of pest species, including insects, rodents, and birds, in the maintenance or potential transmission of microorganisms through the farm environment.

*Stomoxys* flies are ubiquitous blood-feeding pests inhabiting dairy farms across the globe and have been previously implicated as carriers of pathogenic bacteria on dairy farms due to their close association with both bovine manure and cattle hosts (7–12). Adult female flies preferentially oviposit eggs into aged cattle manure, which serves as a protein-, carbohydrate-, and microbe-rich source of nutrition for developing larvae (13–16). As adults, both male and female flies require daily nutritional blood meals from cattle or other mammalian hosts (12, 17). *Stomoxys* flies can excrete ingested bacteria through regurgitation during blood feeding (18, 19) and may further disseminate them into the environment via external carriage or defecation after feeding (11, 20). Defensive behaviors exhibited by livestock during feeding often result in multiple interrupted blood meals (17, 21, 22), increasing the likelihood of *Stomoxys* flies contacting and feeding on multiple bovine hosts. Recently, we performed the first culture-independent, 16S rRNA gene amplicon sequencing-based characterization of bacterial communities associated with adult *Stomoxys* flies and bovine manure samples collected longitudinally in a working dairy facility. Our results showed that the *Stomoxys* microbiota primarily comprises *Enterobacteriaceae*, *Staphylococcaceae*, and other opportunistic pathogenic bacterial taxa (7), which can be readily isolated from both the exogenous and endogenous surfaces of adult flies (8). We found that most bacterial phylotypes sequenced from flies were also present at low relative abundance in manure samples, suggesting that biting flies may acquire bacteria through contact with manure.

*Escherichia coli*, *Klebsiella pneumoniae*, and non-aureus *Staphylococcaceae* are key opportunistic pathogens responsible for environmental bovine mastitis—one of the most significant diseases affecting dairy cattle in the United States (23, 24). These intramammary infections vary in severity, ranging from mild subclinical cases—often indicated by elevated somatic cell counts and reduced milk quality—to severe clinical presentations marked by visible inflammation and abnormal milk production (24). Transmission typically occurs through exposure of the teat to manure or contaminated bedding, making control particularly challenging (1, 24, 25). While some studies have identified potential mastitis virulence factors in *E. coli* and *K. pneumoniae*, host factors largely determine infection outcomes, and isolates from infected cattle are often genomically diverse (26–30). Similarly, a wide range of *Staphylococcaceae*—including *Staphylococcus xylosus* and *Mammaliicoccus sciuri* (formerly *Staphylococcus sciuri*)—have been implicated in environmental mastitis, though their pathogenesis and epidemiology remain poorly understood (23, 31–33). Effective prevention of these opportunistic infections requires a deeper understanding of their ecology, including environmental distribution, genomic diversity, and potential routes of dissemination.

 To address these knowledge gaps, we conducted short-read whole genome sequencing of bacterial isolates primarily obtained from flies and manure samples collected at two dairy facilities in Wisconsin, USA. We performed comparative genomic analyses on 296 bacterial genomes spanning four focal taxa—*E*. *coli*, *K. pneumoniae*, *M. sciuri*, and *Staphylococcus* spp.—all of which are associated with bovine mastitis or zoonotic diseases. To contextualize our findings, we constructed expanded reference genome sets to assess the phylogenetic and functional diversity of isolates from flies, manure, and mastitis cases, alongside bacteria isolated from other hosts and environments.

Our results reveal phylogenetic intermixing between fly- and manure-derived isolates, including the presence of near-identical strains across sample types, supporting a potential role for flies in the dispersal of manure-associated bacteria on dairy farms. Antimicrobial resistance gene (ARG) screening identified clinically relevant extended-spectrum beta-lactamase (ESBL) genes in *Enterobacteriaceae* isolates, along with lincosamide and tetracycline resistance genes in *Staphylococcaceae*, likely carried on mobile genetic elements. Further analysis of *M. sciuri* showed that isolates from flies, manure, and mastitis cases clustered within a distinct clade enriched for xylose metabolism genes (*xylA*, *xylB*), which were absent in other *M. sciuri* lineages. Collectively, these results highlight the interconnectedness of fly and manure microbiomes and suggest a potential role for flies in the transmission of mastitis-associated pathogens within dairy environments.

## RESULTS

### Development of a bacterial isolate collection

In three prior studies, we established a collection of bacterial isolates derived from flies and manure collected at two focal dairy farms in south-central Wisconsin (7, 8, 34). This collection comprises approximately 1,200 isolates, from which 80 manure-derived and 208 fly-derived isolates were selected for short-read whole genome sequencing in the present study. Additionally, we sequenced eight clinical mastitis isolates—one *E. coli*, six *K. pneumoniae*, and one *Staphylococcus haemolyticus*—obtained from mastitic cows housed at the same facilities and archived by the Wisconsin Veterinary Diagnostic Laboratory in Madison, WI, USA.

### Collection of draft genomes from *Stomoxys* flies and the barn environment

**Fig 1:**
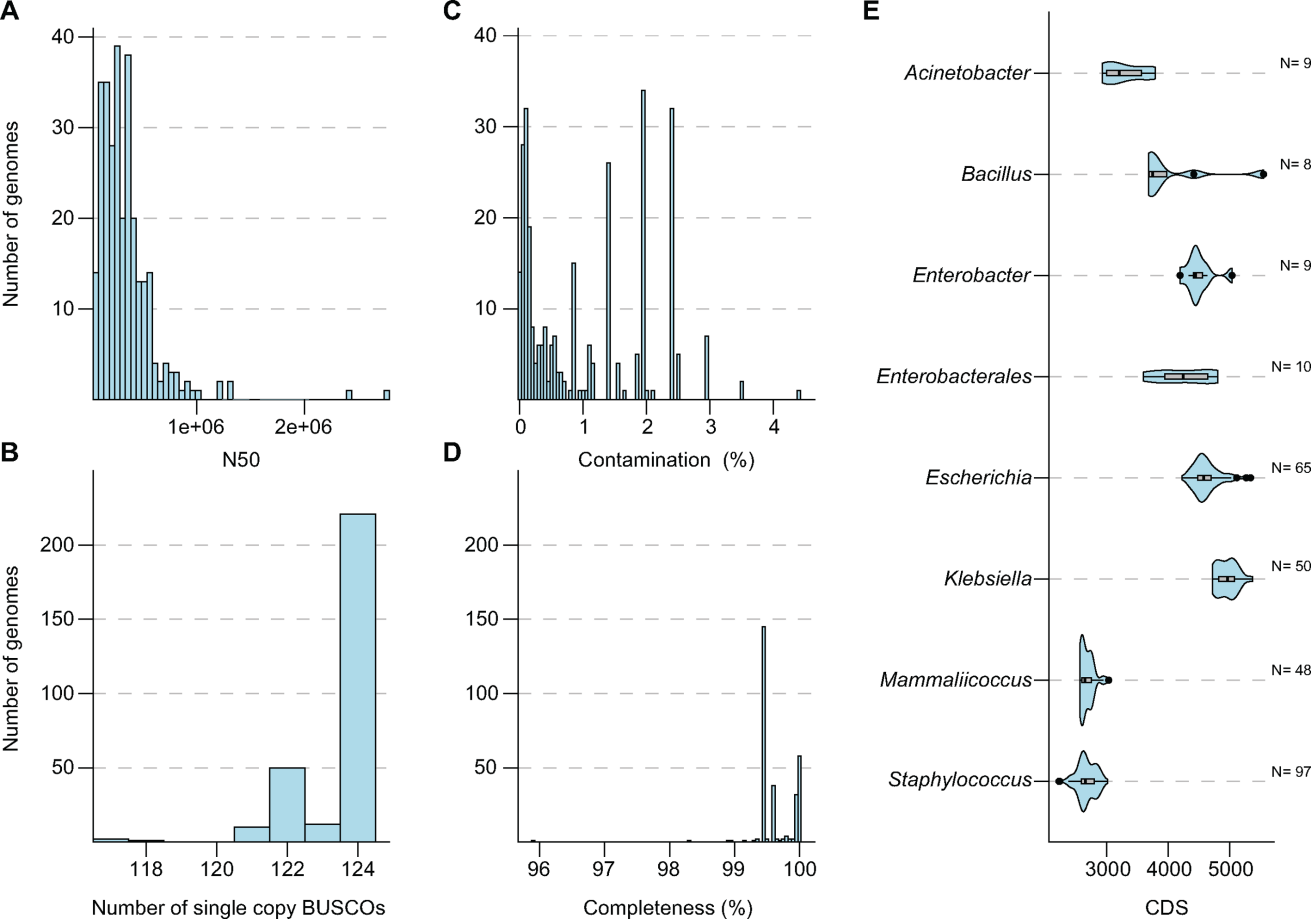
Genome assembly statistics. (**A**) Histogram depicting the distribution of N50 contig lengths across sequenced genomes. (**B**) Histogram depicting the number of BUSCO (Benchmarking Universal Single-Copy Orthologs) detected across sequenced genomes. (C and D) Histograms depicting the calculated contamination (**C**) and completeness (**D**) levels across sequenced genomes. (**E**) Violin plots showing the distribution of CDS (coding sequences) across sequenced taxonomic groups. *N* represents the number of genomes within each taxonomic group.

A total of 296 genomes were sequenced in this study, comprising 208 fly-, 80 manure-, and eight mastitis-derived isolates (Table S1). Draft genome assemblies were evaluated using Quast (35), yielding a median N50 of 285,278 base pairs (bp) across all isolates (Fig. 1A). Genome completeness was assessed using BUSCO (Benchmarking Universal Single-Copy Orthologs), which identified all 124 expected orthologs in 221 assemblies, with the remaining genomes containing between 118 and 123 orthologs (Fig. 1B) (36). CheckM analysis, using a full reference tree, estimated a median genome completeness of 99.45% (range: 95.91%–100%) and a median contamination level of 0.735% (range: 0%–4.38%) (Fig. 1C and D). Taxonomic classification via PubMLST ribosomal Multilocus Sequence Typing (rMLST) identified *E. coli*, *K. pneumoniae*, and species of *Staphylococcus* and *Mammaliicoccus* as the most prevalent taxa in the data set (Table S1; Fig. 1E). All assembled genomes were subsequently screened for antimicrobial resistance genes (ARGs), virulence factors (VFs), and plasmid replicon genes, as appropriate (Table S2) (37–41).

### Analysis of sequenced *E. coli* isolates

**Fig 2:**
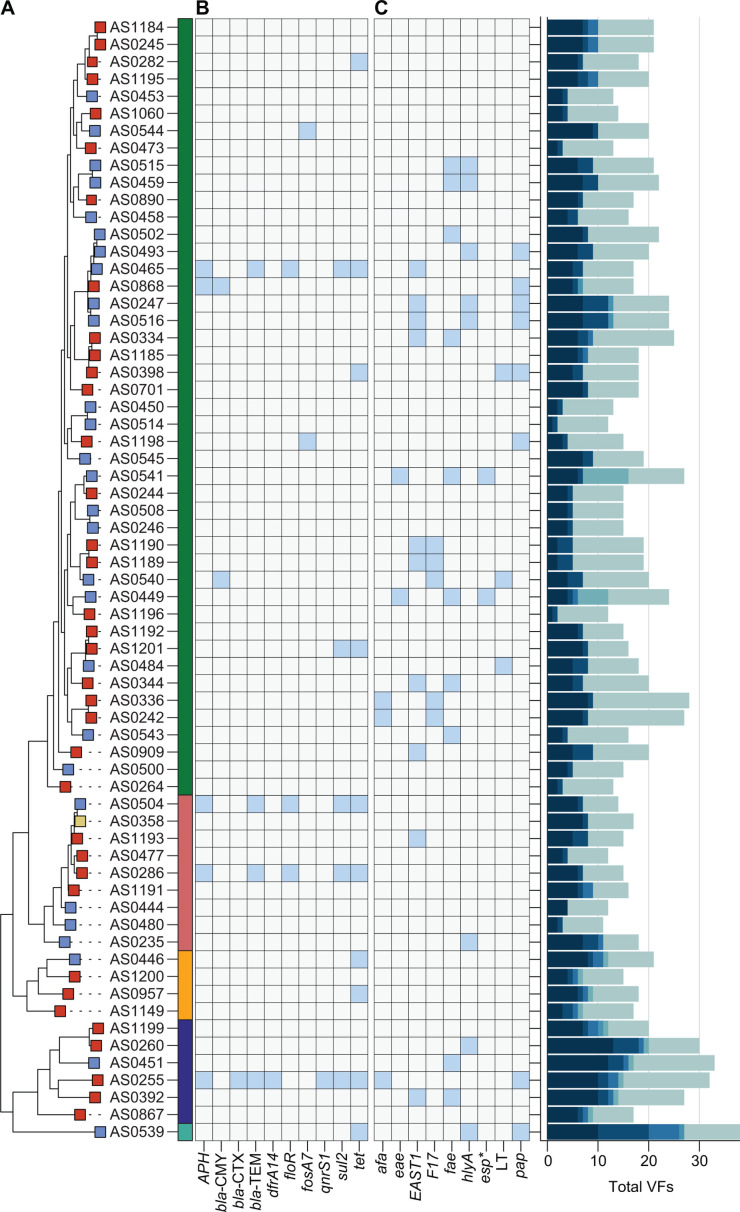
Phylogenetic intermixing of sequenced *E. coli* strains. (**A**) Midpoint rooted maximum-likelihood phylogenetic tree based on a core gene alignment of sequenced *E. coli* isolates. The rectangular node to the left of the strain name indicates the origin of each isolate as follows: flies (red), manure (blue), and mastitis (yellow). The colored bar to the right of the strain name indicates the MASH phylogroup of each isolate: B1 (green, top), A (light red), E (orange), D (dark blue), and B2 (cyan, bottom). (**B**) Heatmap depicting the presence-absence matrix (blue indicates presence) of select ARGs (Resfinder) and VFs (VirulenceFinder). *esp refers to strains positive for either *espA*, *espB*, or *espJ*. (**C**) Bar graph showing the total number of VFs detected in each strain. Stacked colors represent counts of different classes of VFs as follows from light to dark: adhesion, effector delivery system (EFD), invasion, iron uptake, toxin, and other VFs.

We performed a phylogenetic analysis of the 65 *E. coli* isolates sequenced in this study, which included 36 fly-*,* 28 manure-, and one mastitis-derived *E. coli* isolates. A maximum-likelihood phylogenetic tree was constructed based on a core genome alignment to show the local population structure of sequenced *E. coli* isolates (Fig. 2A). Bacterial isolates clustered based on calculated MASH phylogroups (42), with the majority of isolates belonging to phylogroup B1 (Fig. 2A). Conversely, only a single isolate (manure-derived) in the collection belonged to phylogroup B2, and a small minority of isolates belonged to phylogroup D. We observed phylogenetic intermixing between fly- and manure-derived *E. coli* isolates on the phylogeny, which included two sets of fly and manure isolate pairs with the same sequence type (ST). The single mastitis-derived *E. coli* isolate also shared the same sequence type (ST2) as the fly and manure isolates, although all three isolates had different predicted serotypes (Table S3).

*E. coli* draft assemblies were screened for the presence of acquired ARGs and VFs using the ResFinder and VirulenceFinder databases, respectively (40, 41). Acquired ARG carriage was overall rare throughout the collection; however, we did find both fly- and manure-derived *E. coli* isolates that harbored extended-spectrum beta-lactamase genes (ESBLs), including *bla*-CTX, *bla*-CMY, and *bla*-TEM (Fig. 2B). Carriage of ESBLs was not confined to a specific phylogroup. Virulence factor carriage was mixed throughout the phylogenetic tree, with fly and manure isolates carrying a variety of adhesins, toxins, and iron uptake genes (Fig. 2C). This notably included four fly-derived *E. coli* isolates and one manure-derived isolate encoding genes related to yersiniabactin siderophore production (*irp*, *fyuA*, *ybt*) (Table S2; Table S3). None of the sequenced isolates from either manure or flies carried Shiga toxin (*stx*) genes. However, two manure-derived isolates belonging to phylogroup B1 encoded *esp* genes (*espA*, *espB*, and *espJ*), which are associated with the LEE4 (locus of enterocyte effacement) pathogenicity island. Additionally, several other isolates carried the *espY2* gene, which encodes a type III secretion system (T3SS) effector-like protein (Table S3) (43, 44). Adhesive fimbriae genes identified among the sequenced isolates included F17 fimbriae, *pap* fimbriae, and Afa/Dr family (*afa*) adhesins.

**Fig 3:**
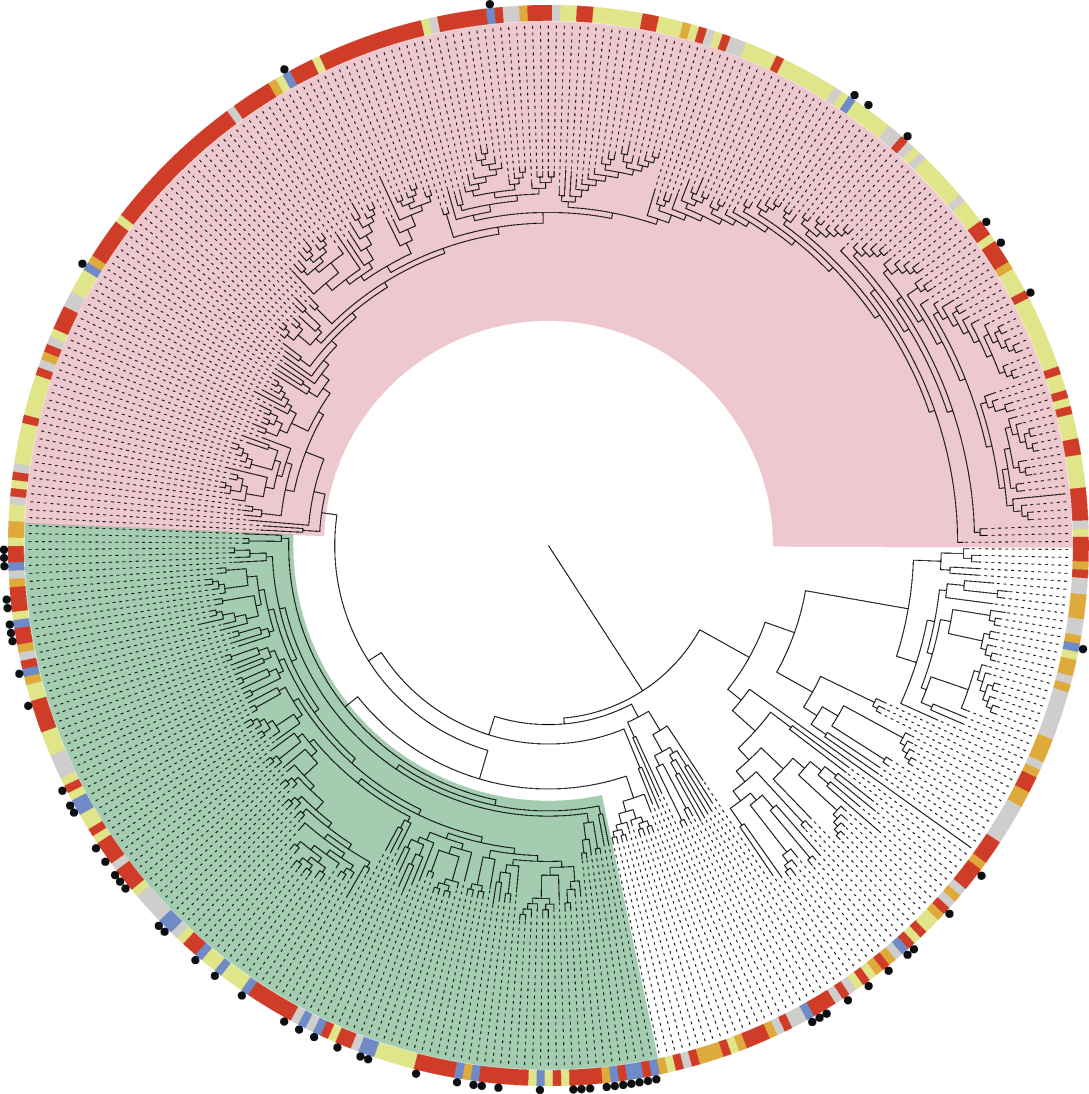
*E. coli* population structure and phylogenetic placement of sequenced *E. coli* strains. A midpoint-rooted maximum-likelihood phylogenetic tree was constructed based on a core gene alignment of 410 *E. coli* genomes. Isolates sequenced in this study are marked with a black circle. The node color indicates the origin of each isolate as follows: flies (red), manure (blue), mastitis (yellow), other host (nonclinical) (gray), and other host (clinical) (orange). The phylogroup A *E. coli* clade is highlighted in red; the phylogroup B1 *E. coli* clade is highlighted in green.

To place the sequenced *E. coli* isolates within a broader evolutionary context, we constructed a maximum-likelihood phylogenetic tree based on a core genome alignment of 410 *E. coli* genomes (Fig. 3). This data set included 126 publicly available fly-derived genomes and 121 mastitis-derived genomes. According to NCBI records, the fly-derived bacterial genomes were primarily isolated from *Musca domestica* (Family: Muscidae) or unidentified fly species, with a subset originating from *Chrysomya* spp. (Family: Calliphoridae) (Table S4). These fly-derived isolates were distributed across the entire *E. coli* phylogeny, with the majority belonging to phylogroup A (*n* = 89), followed by phylogroup B1 (*n* = 33). A similar distribution was observed among mastitis-derived genomes, with 84 assigned to phylogroup A and 28 to phylogroup B1 based on Mash analysis. Pangenome analysis further revealed that genome clustering was largely driven by phylogroup, as reflected in gene content similarity (Fig. S1).

### Analysis of sequenced *K. pneumoniae* isolates

**Fig 4:**
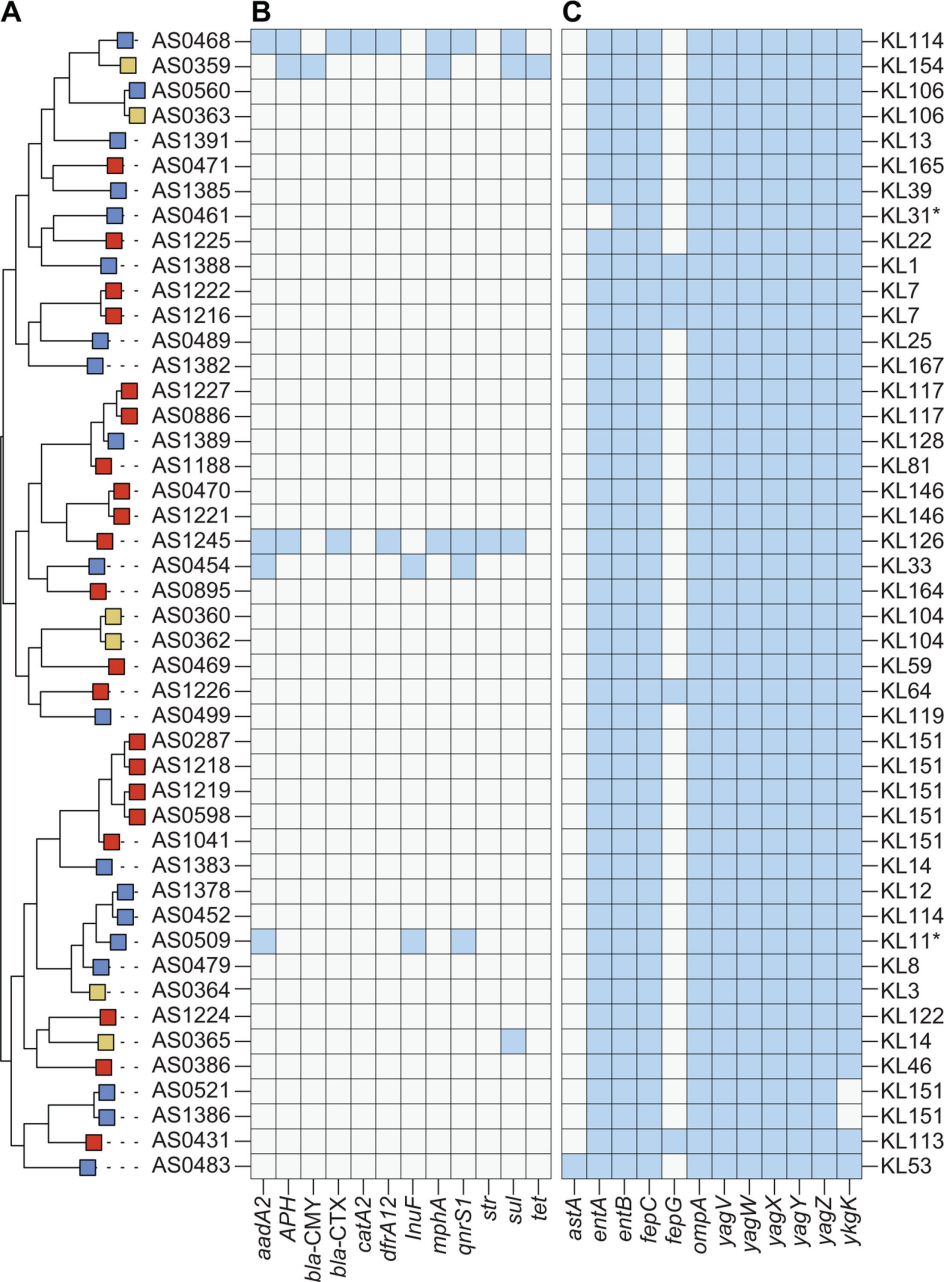
Phylogenetic intermixing of sequenced *K. pneumoniae* strains. (**A**) Midpoint-rooted maximum-likelihood phylogenetic tree based on a core gene alignment of sequenced *K. pneumoniae* isolates. The rectangular node to the left of the strain name indicates the origin of each isolate as follows: flies (red), manure (blue), and mastitis (yellow). (B and C) Heatmap depicting the presence-absence matrix (blue indicates presence) of select ARGs (Resfinder) (**B**) and VFs (VFDB) (**C**). The predicted capsular polysaccharide type is provided to the right of the heatmaps.

We constructed a maximum-likelihood phylogenetic tree based on a core genome alignment of 46 sequenced *K. pneumoniae* isolates—21 fly-, 19 manure-, and six mastitis-derived—which revealed three distinct lineages with intermixed sample origins within each lineage (Fig. 4A). Although carriage of acquired ARGs was generally rare, two fly- and two manure-derived isolates carried ESBL genes (Fig. 4B). In contrast to *E. coli*, *K. pneumoniae* isolates exhibited limited variation in VFs, and none harbored gene clusters associated with hypervirulence (*ybt*, *clb, iuc*, *iro*, and *rmp*) (Fig. 4C; Table S5) (45). *In silico* analysis of capsular polysaccharide (CPS) loci identified 33 distinct K-locus types, with only KL14 and KL151 shared between fly- and manure-derived isolates. However, in both cases, the corresponding isolates belonged to different STs (Fig. 4C; Table S5). Notably, KL106 was detected in both a manure- and a mastitis-derived isolate, which clustered together in the phylogeny and shared the same ST (ST101).

**Fig 5:**
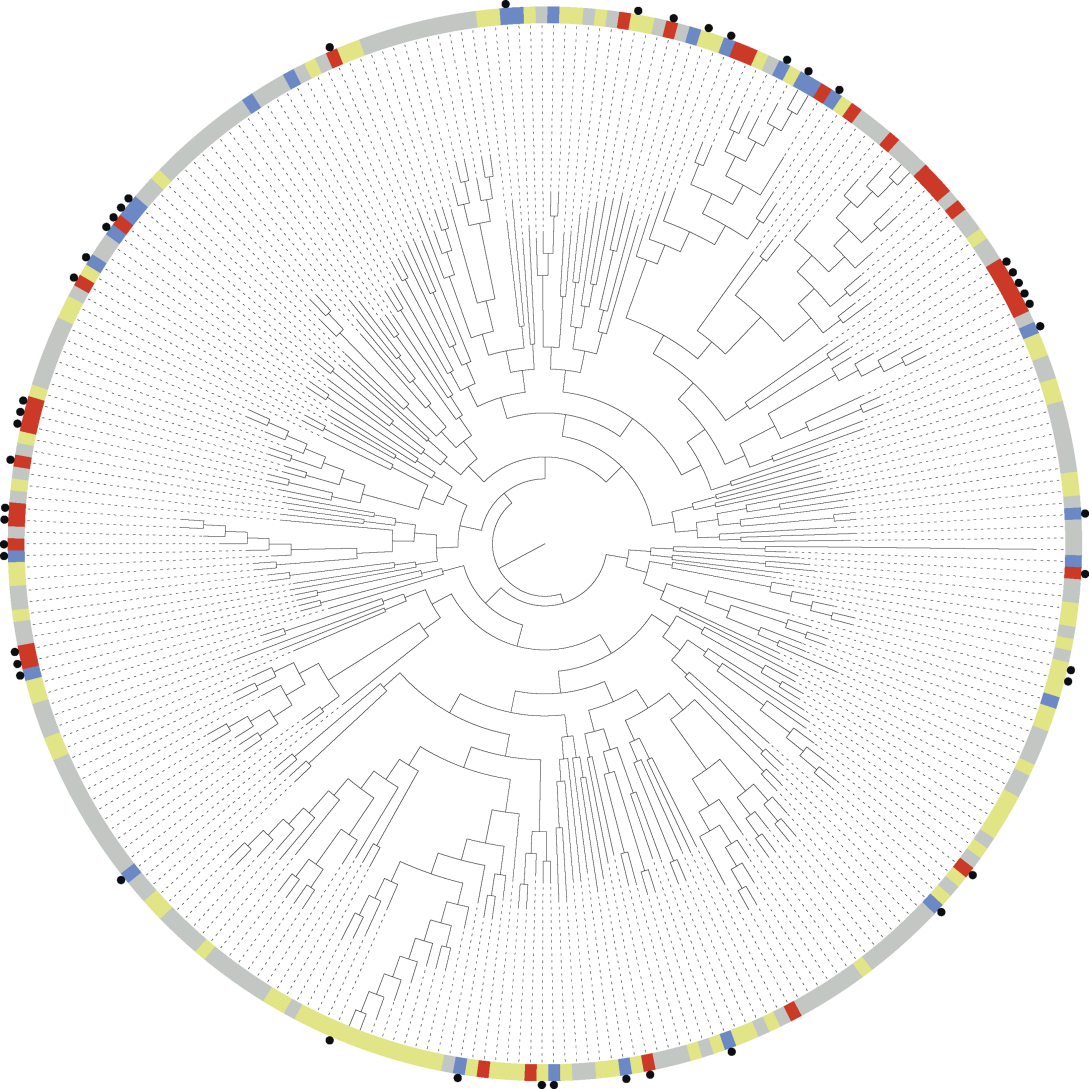
*K. pneumoniae* population structure and phylogenetic placement of sequenced *K. pneumoniae* strains. A midpoint-rooted maximum-likelihood phylogenetic tree was constructed based on a core gene alignment of 281 *K*. *pneumoniae* genomes. Isolates sequenced in this study are marked with a black circle. The node color indicates the origin of each isolate as follows: flies (red), manure (blue), mastitis (yellow), and other (gray).

To further contextualize these findings, we assembled a reference genome set of 281 *K*. *pneumoniae* isolates, including 80 mastitis- and 13 fly-derived genomes (Table S4). The resulting phylogenetic tree confirmed that flies harbor diverse *K. pneumoniae* lineages (Fig. 5). Despite the presence of deeply branching lineages, pangenome analysis revealed no clear clustering of isolates or lineages based on gene content (Fig. S2).

### Analysis of other sequenced *Enterobacteriaceae*

In addition to the focal taxa, we sequenced a limited number of other *Enterobacteriaceae* isolates, including *Citrobacter tructae* (*n* = 1), *Enterobacter asburiae* (*n* = 1), *Enterobacter hormaechei* (*n* = 7), *Enterobacter kobei* (*n* = 1), *Klebsiella aerogenes* (*n* = 3), *Klebsiella michiganensis* (*n* = 1), *Kosakonia sacchari* (*n* = 1), *Proteus terrae* (*n* = 1), *Pseudescherichia vulneris* (*n* = 1), *Raoultella ornithinolytica* (*n* = 1), *Salmonella enterica* (*n* = 1), *Serratia marcescens* (*n* = 1), *Serratia nevei* (*n* = 1), and *Siccibacter turicensis* (*n* = 2) (Table S1). Screening for ARGs revealed that one *E. hormaechei* isolate (AS0467) carried the ESBL genes *CTX-M-15* and *TEM-1B* (Table S2).

### Analysis of sequenced *Staphylococcaceae* isolates

A total of 145 *Staphylococcaceae* isolates were sequenced in this study, including 127 fly-derived isolates, 17 manure-derived isolates, and one mastitis-derived isolate. These were classified into 13 *Staphylococcus* species and two *Mammaliicoccus* species, including *Mammaliicoccus lentus* (*n* = 2), *M. sciuri* (*n* = 46), *Staphylococcus arlettae* (*n* = 5), *S. caeli* (*n* = 2), *S. chromogenes* (*n* = 2), *S. equorum* (*n* = 1), *S. gallinarum* (*n* = 8), *S. haemolyticus* (*n* = 1), *S. pasteuri* (*n* = 1), *S. pseudoxylosus* (*n* = 8), *S. saprophyticus* (*n* = 2), *S. shinii* (*n* = 21), *S. succinus* (*n* = 3), *S. ureilyticus* (*n* = 1), and *S. xylosus* (*n* = 42) (Table S1). *M. sciuri* (formerly *S. sciuri*) was the most prevalent species, with 35 fly- and 11 manure-derived isolates.

**Fig 6:**
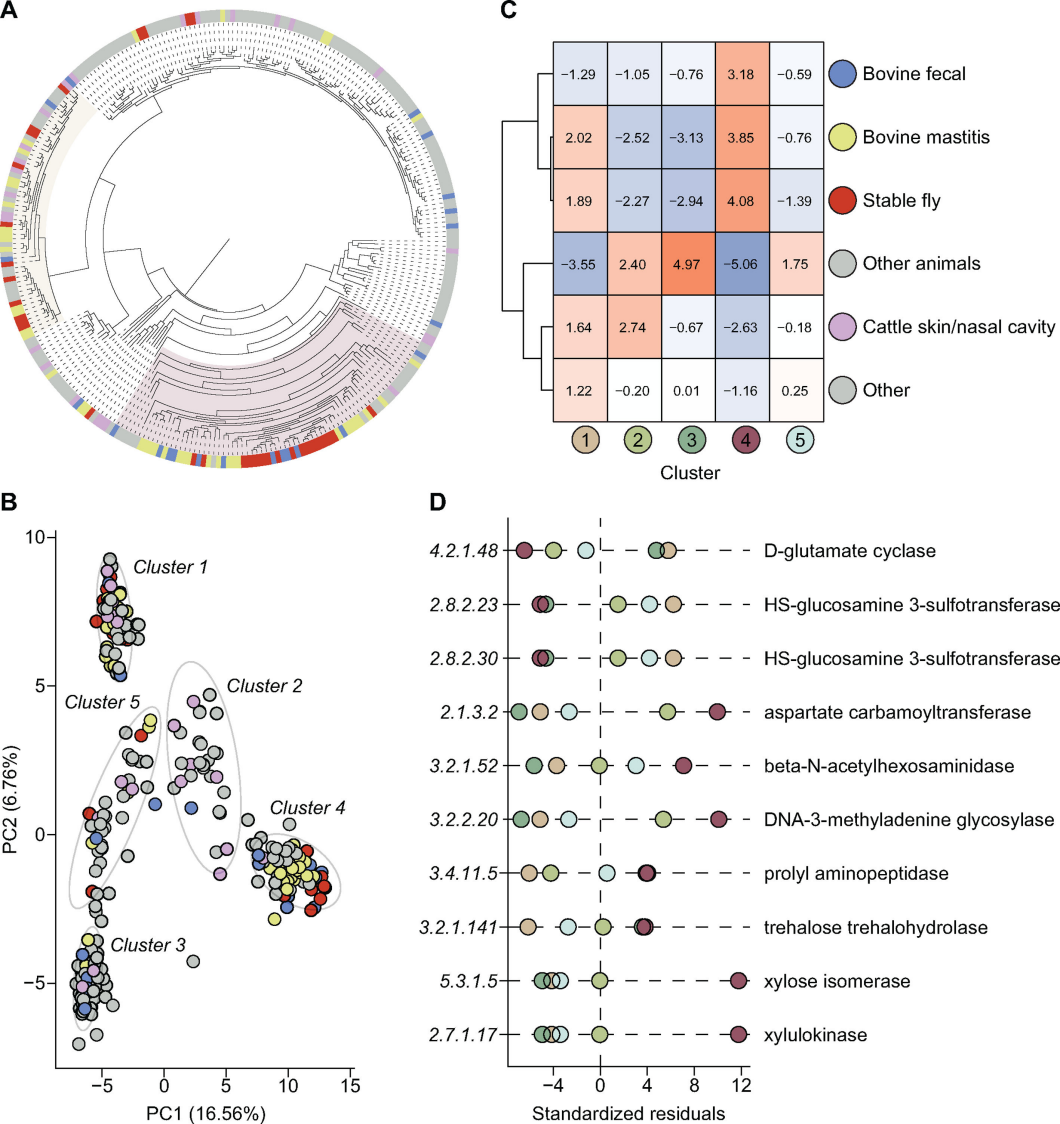
*M. sciuri* genomic analysis. (**A**) Midpoint-rooted maximum-likelihood phylogenetic tree based on a core gene alignment of 281 *M*. *sciuri* genomes, including the 35 fly- and 11 manure-derived strains sequenced in this study. Sample origins are colored as follows: flies (red), manure or cattle gastrointestinal tract (blue), bovine mastitis (yellow), cattle skin or nasal cavity (purple), and other (gray). The two fly-associated clusters of interest are highlighted respectively on the tree. (**B**) PCA clustering of genomes based on gene content as determined by Panaroo. Ellipses were calculated with a 95% confidence interval for the five clusters identified through k-means analysis. (**C**) Heatmap of Chi-squared standardized residuals (10,000 Monte Carlo simulations) showing the association of isolates from different sources with each k-means cluster. The sample origins were further hierarchically clustered based on Euclidean distances, as shown by the dendrogram. (**D**) Dot plot of Chi-squared standardized residuals (10,000 Monte Carlo simulations) showing the association of select ECs with *M. sciuri* k-means clusters. The dot plot shows KEGG ECs for clusters 1 and 4 with a calculated standardized residual value greater than 3.5 and a prevalence greater than 80%. The full list of residual values for all ECs is available in Table S6.

To explore the phylogenetic diversity of *M. sciuri*, we compiled a reference genome set of 235 publicly available genomes (after quality filtering) and constructed a core genome phylogeny based on 2,037 orthologs. Most fly-derived isolates clustered within two major clades (Fig. 6A). This tree was compared to an outgroup-rooted phylogeny based on seven housekeeping genes from the *M. sciuri* MLST scheme (*ack*, *aroE*, *ftsZ*, *glpK*, *gmk*, *pta1*, and *tpiA*) (Fig. S3). (46). Pangenome analysis revealed an open pangenome with high accessory gene content (Fig. S4). Using k-means clustering, we identified five gene content-based clusters, with clusters 1 and 4 aligning closely with fly-associated phylogenetic clades (Fig. 6B; Fig. S3).

A Chi-square test with Monte Carlo simulations revealed a significant association between cluster membership and isolation source (*P* = 9.999e-05) (Fig. 6C). Standardized residuals indicated that fly-, manure-, and mastitis-derived isolates were strongly associated with cluster 4 (*R* > 3), while skin/nasal-derived isolates were enriched in cluster 2 (*R* = 2.74). Cluster 1 also showed associations with both mastitis- (*R* = 2.02) and fly-derived isolates (*R* = 1.89). Functional annotation using eggNOG-mapper identified KEGG (Kyoto Encyclopedia of Genes and Genomes) Orthologs (KOs) and Enzyme Commission (EC) numbers (47–49). A second Chi-square test on EC group counts (*P* = 9.999e-05) revealed 10 ECs significantly enriched (*R* > 3.5, prevalence >80%) in fly-associated clusters (Fig. 6D; Table S6). Notably, ECs related to [heparan sulfate]-glucosamine 3-sulfotransferase were exclusive to cluster 1, while ECs involved in xylose metabolism were highly enriched in cluster 4 (Fig. 6D; Table S6).

Phylogenetic analysis across the *Staphylococcaceae* family revealed closely related fly- and manure-derived isolates in *M. sciuri*, *S. arlettae*, *S. gallinarum*, and *S. xylosus* (Fig. 6A; Fig.S5-S9). Pairwise genome alignments using MUMmer4 identified several highly similar isolate pairs (Table 1; Table S7) (50). Three *S*. *gallinarum* isolate pairs shared >99.99% aligned bases, with only 12–351 unaligned base pairs. Other isolate pairs showed 95.85%–99.99% alignment. For the closest *M. sciuri* pair (AS0296–AS0565), four unique contigs were identified in the fly-derived isolate, including a 3,016 bp contig encoding plasmid *rep* and *pre* genes (Table S7). Similarly, the *S. arlettae* pair (AS0200–AS0572) included four unique contigs in the fly-derived isolate, one of which encoded *pre*, *rep*, and *tet* genes on a 4,551 bp contig.

**TABLE 1:** MUMmer4 alignment summary of fly and manure genome pairs clustered together on the core genome phylogenies.^*a*^

	Fly isolate	Bases aligned	Bases unaligned	Manure isolate	Bases aligned	Bases unaligned
*M. sciuri*	AS0296	99.7454%	6,815	AS0565	99.9495%	1,350
	AS1298	98.7064%	33,462	AS0567	98.0334%	51,283
	AS0650	95.2483%	127,306	AS1395	98.5790%	36,823
	AS0913	96.5620%	89,859	AS0579	95.8520%	109,361
*S. arlettae*	AS0200	99.4203%	14,808	AS0572	99.9797%	515
*S. gallinarum*	AS0915	99.9996%	12	AS0571	99.9996%	31
	AS0915	99.9962%	112	AS0564	99.9951%	144
	AS1286	99.9880%	351	AS0564	99.9961%	113
	AS1336	98.0958%	56,631	AS0564	99.9860%	408
*S. xylosus*	AS1331	98.5428%	40,873	AS0569	98.3096%	47,531

^*a*^ Manure isolates AS0571 and AS0569 and fly isolate AS1286 were obtained from samples collected from the Dairy Cattle Center (DCC), a smaller satellite farm of the main sample collection site. Detailed alignment statistics are available in Table S7.

### Similarity of ARG-encoding plasmid sequences among diverse *Staphylococcaceae* isolates

**Fig 7:**
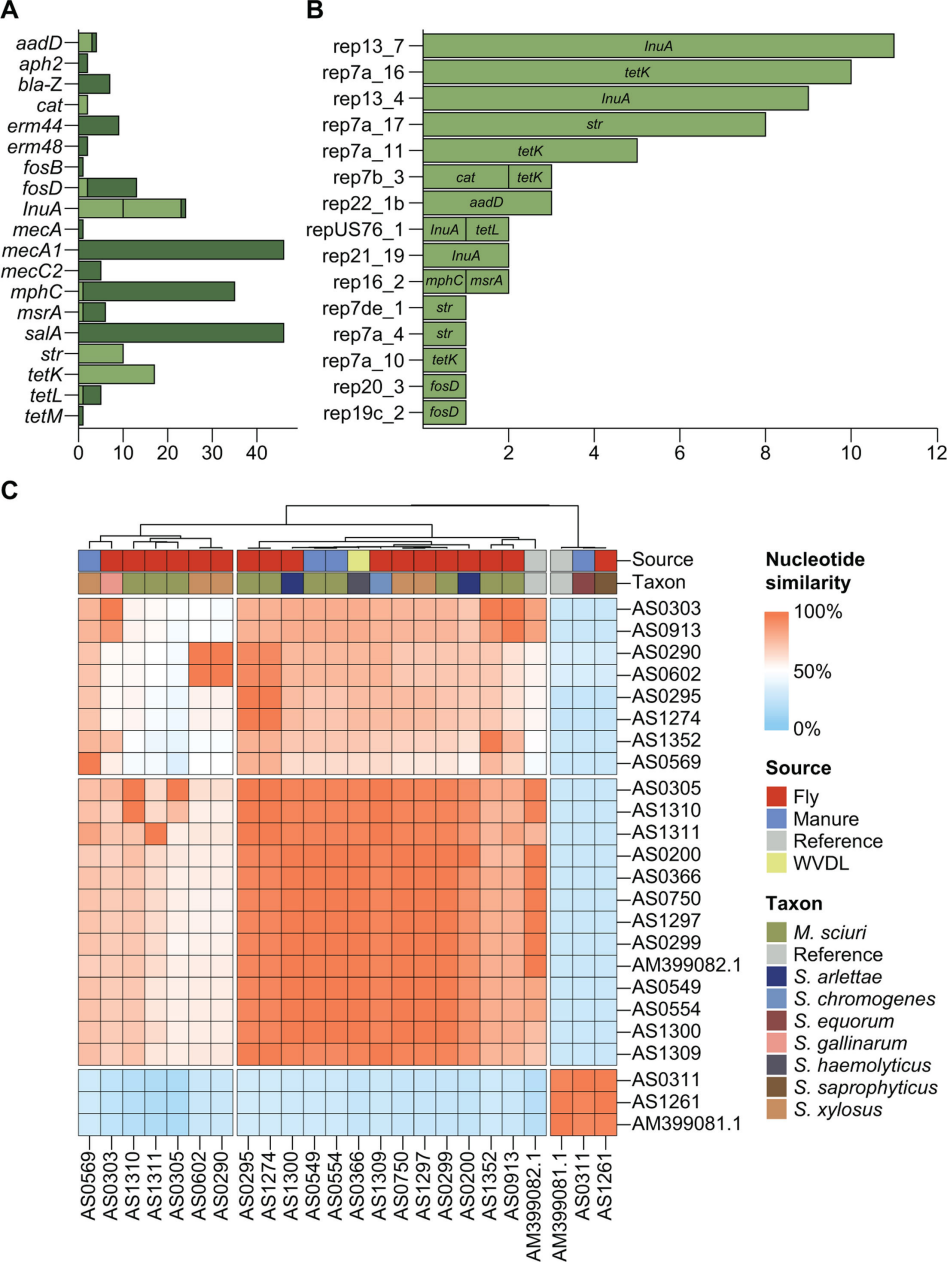
*Staphylococcaceae* ARG analysis. (**A**) Bar plot showing the counts of different ARGs found either on the same contig as a plasmid *rep* gene (light green) or on a contig without a *rep* gene (dark green). (**B**) Bar plot showing the counts of ARGs found on the same contig as different plasmid replicon types, as identified by PlasmidFinder. (**C**) Heatmap depicting the global percent nucleotide similarity of plasmid-associated contigs encoding the *lnuA* gene. Red indicates a greater percent nucleotide similarity, while blue indicates a lower percent similarity. The associated strain name is labeled on both the rows and columns, with AM399082.1 and AM399081.1 included as reference plasmids. Both rows and columns were hierarchically clustered based on Euclidean distance, with only the column dendrogram shown. The origin and taxonomic identification of the strains are marked below the dendrogram. Four strains (AS0311, AS0554, AS0569, and AS1261) were isolated from samples collected from the Dairy Cattle Center (DCC), a smaller satellite farm of the main sample collection site.

All sequenced *Staphylococcaceae* genomes were screened for both plasmid *rep* genes and ARGs to identify potential plasmid-borne ARGs shared among isolates. Of the 19 ARGs detected, nine were found exclusively on contigs lacking a *rep* gene, suggesting either chromosomal localization or presence on larger plasmids not fully resolved by short-read sequencing. In contrast, *lnuA* and *tetK* were the most frequently observed ARGs co-located with plasmid *rep* genes, typically appearing as the sole ARG on their respective contigs (Fig. 7A; Table S8).

Three plasmid replicon types—Rep13_7, Rep13_4, and Rep21_19—were associated with contigs encoding a single copy of *lnuA*, with contig lengths ranging from 2,126 to 5,110 bp (Fig. 7B, Fig. S10). Pairwise sequence alignments of all 22 *lnuA*-containing contigs, along with three reference plasmids, were performed using MUMmer4-MobMess (50, 51). Global average nucleotide identity (ANI) among contigs ranged from 13.27% to 100%, and k-means clustering revealed three distinct groups, with some intermixing between Rep13_7 and Rep13_4 plasmids (Fig. 7C; Table S8). Notably, a 2,848 bp contig containing *lnu*A and Rep13_7 was shared with 100% ANI across seven isolates, including one fly-derived *M. sciuri* (AS0299), two manure-derived *M. sciuri* (AS0549, AS0554), two fly-derived *S. xylosus* (AS0750, AS1297), one fly-derived *S. arlettae* (AS1300), and one mastitis-derived *S. haemolyticus* (AS0366).

A similar analysis of *tetK*-associated plasmid contigs revealed ANI values ranging from 76.60% to 100%. Thirteen of the 16 *tetK* contigs clustered with two full reference plasmids, exhibiting high sequence similarity (97.30% to 100%; median 99.76%) and comparable lengths (4,439–4,612 bp) (Fig. S11-S12).

## DISCUSSION

The primary objective of this study was to investigate the potential ecological transmission of manure-associated bacterial isolates to hematophagous *Stomoxys* flies. Populations of *Stomoxys* in livestock facilities are sustained by the continuous availability of mammalian hosts and manure, both of which serve as critical developmental and nutritional resources for these biting muscid flies (12–16). Manure also acts as a significant reservoir for human and bovine pathogens, including opportunistic members of the *Enterobacteriaceae* and *Staphylococcaceae* families, which are known to cause udder inflammation (bovine mastitis) in lactating cattle (1).

Both culture-based and amplicon sequencing studies have shown that the *Stomoxys* microbiota is largely composed of opportunistic pathogens (7–10). However, genomic studies are still needed to confirm the connection between biting flies and manure-associated bacterial reservoirs. This study, therefore, aimed to assess the genomic diversity of fly-derived bacteria in comparison to isolates associated with cattle hosts. For *E. coli*, we found that fly- and manure-derived isolates were interspersed throughout the reconstructed phylogenetic tree, indicating no evidence of *Stomoxys*-exclusive *E. coli* lineages in the sequenced samples. Instead, most isolates from both sources belonged to phylogroups A and B1, which are commonly associated with a commensal niche in the bovine digestive tract (52–55).

Notably, a minority of isolates carried Afa/Dr adhesin genes, the primary PCR diagnostic markers for the diffusely adherent *E. coli* (DAEC) diarrheagenic pathotype (56). Additionally, a subset of fly-derived isolates harbored F17 family fimbriae, which facilitate bacterial adherence to bovine intestinal mucosa and have been linked to enteric and septicemic infections in calves (57, 58). However, these isolates did not encode any known heat-labile toxins (LT), which are key virulence factors of enterotoxigenic *E. coli* (ETEC) (59). Interestingly, a subset of phylogroup A and B1 isolates encoded the high pathogenicity island (HPI)—a biosynthetic gene cluster responsible for producing the siderophore yersiniabactin (Ybt), a determinant of high virulence originally identified in *Yersinia enterocolitica* (60, 61). The single mastitis-derived *E. coli* isolate in our collection shared the same ST as both a manure- and a fly-derived isolate and notably lacked many identifiable VFs. This finding aligns with previous studies suggesting that mastitis-causing *E. coli* are opportunistic environmental strains rather than pathogens with distinct virulence profiles (29, 62). When compared to a broader reference phylogeny, most published mastitis-associated *E. coli* genomes were also assigned to phylogroups A or B1, further supporting the link between bovine mastitis and gastrointestinal *E. coli* populations.

We also compared our sequenced *E. coli* isolates to previously published genomes from other fly taxa. Several studies have used whole genome sequencing to analyze *E. coli* from nonbiting flies, including *M. domestica* (63, 64), *Chrysomya* spp. (65), and unidentified fly taxa (66–70). However, these samples were collected from non-dairy environments, such as swine farms, poultry farms, or hospitals. Inclusion of these isolates in our reconstructed phylogenetic tree revealed substantial diversity in fly-derived *E. coli*, likely reflecting acquisition through environmental interactions specific to each fly’s habitat.

Sequenced *K. pneumoniae* isolates formed three distinct phylogenetic clades, with intermixing of fly-, manure-, and mastitis-derived isolates. Aside from capsule type, few differences in identified virulence factors were observed among isolates, including those derived from mastitis cases. Both the capsular polysaccharide (CPS) and the lipopolysaccharide (LPS) O antigen are key virulence determinants in *K. pneumoniae*, playing critical roles in mucosal colonization and evasion of host immune responses (71). Among the isolates, we identified a manure-derived strain encoding the K1 capsule type, a capsule variant commonly associated with human pathogenic *K. pneumoniae* (72). While the polysaccharide capsule is also thought to facilitate invasion of the bovine mammary gland, previous studies have reported considerable diversity in CPS and LPS types among mastitis-derived isolates (30, 73). Our species-wide phylogenetic analysis further revealed that fly-, manure-, and mastitis-derived isolates were intermixed with human-derived strains, indicating no clear host-specific clustering. Notably, fly-associated isolates were distributed across multiple subclades, suggesting that muscid flies can harbor a diverse array of *K. pneumoniae* lineages. Pangenome analysis showed no large-scale clustering of isolates based on gene content, which may reflect a large accessory genome and frequent horizontal gene transfer among divergent *K. pneumoniae* strains. Alternatively, this lack of clustering could be due to limited genomic representation from environmental and non-human sources, which currently constitute only a small fraction of all sequenced *K. pneumoniae* genomes (74).

A major limitation hindering further analysis of many non-aureus *Staphylococcaceae* species is the lack of publicly available genomes for comparative studies. Despite their role as the most frequent causative agents of subclinical bovine mastitis (23), the genomic and functional diversity of coagulase-negative *Staphylococcaceae* remains poorly understood. In this study, we conducted a comparative analysis of *M. sciuri* (formerly *S. sciuri*), the most commonly sequenced *Staphylococcaceae* species in our data set. Inclusion of all publicly available *M. sciuri* genomes revealed strong co-clustering of fly-, manure-, and mastitis-derived isolates based on gene content. This manure-associated cluster also corresponded to a distinct clade in the species-wide phylogeny constructed from core genome alignments.

While the xylan hydrolase (*xynB*) gene was present in nearly all *M. sciuri* genomes, those within the manure-associated cluster showed significant enrichment for metabolic genes involved in xylose metabolism, including *xylA* (conversion of D-xylose into D-xylulose) and *xylB* (conversion of D-xylulose to D-xylulose-5-phosphate). These genes were rare or absent in other *M. sciuri* clusters. Xylose is a key component of hemicellulose, a major plant fiber found in ruminant feces (75–77), and the ability to metabolize it may confer a competitive advantage in the bovine gastrointestinal (fecal) niche. These findings provide further evidence that manure-associated *M. sciuri* strains may act as both causative agents of mastitis and prominent members of the *Stomoxys* microbiome. While our results offer novel insights into potential niche differentiation in *M. sciuri*, further research is needed to validate these hypotheses. This includes large-scale, parallel sampling of skin and manure microbiota, as well as experimental validation of metabolic potential and colonization ability. Such experiments were not conducted in the present study due to the limited availability of *M. sciuri* isolates from diverse hosts and environments. Additionally, colonization studies using a fly model would be necessary to determine whether specific strains are better adapted to colonize the fly gut, or whether the observed phylogenetic patterns simply reflect environmental exposure to cattle feces.

In this study, we primarily sequenced isolates from a large free-stall barn system, with a subset obtained from a nearby, smaller tie-stall barn. Although these barns are geographically distinct, cattle are frequently moved between them as part of an integrated herd management system (7). Pairwise genome comparisons of phylogenetically clustered isolates revealed near-identical genomes (up to 99.996% bp alignment) between fly- and manure-derived isolates. This was particularly striking in a trio of *S. gallinarum* isolates—including one from manure at the satellite facility—which differed by fewer than 100 unaligned bp across their entire draft genomes. In several cases, genomic differences were primarily due to the presence or absence of whole contigs containing *rep* genes or other plasmid-associated elements, suggesting that sequence divergence was driven by mobile genetic elements. Collectively, these findings indicate that *Staphylococcus* strains and their associated mobile elements can circulate within the dairy barn environment via *Stomoxys* flies, with the potential for cross-facility dissemination.

A second major goal of this study was to assess the potential dispersal of ARGs across the farm environment. In *Enterobacteriaceae*, ESBLs—often encoded on plasmids or other mobile elements—confer resistance to clinically important beta-lactam antibiotics used in both human and veterinary medicine (78). We detected ESBL genes (*bla*-TEM, *bla*-CTX, *bla*-CMY) in *Enterobacter*, *Klebsiella*, and *E. coli* genomes; however, only four of these were located on the same contig as a plasmid replicon. We previously reported that these strains exhibited cephalosporin resistance, which could be horizontally transferred to naïve strains via conjugation *in vivo* (34). The current results likely represent a limitation of short-read sequencing, which is not well-suited for assembling large or complex plasmids (79). In contrast, *Staphylococcus* species often carry ARGs on small plasmids, which are more effectively captured by short-read sequencing (79–81). The *lnuA* and *tetK* genes were the most frequently identified ARGs on contigs containing plasmid *rep* genes, and these showed strong structural similarity to known reference plasmids. Analysis of these predicted plasmids revealed near-identical sequences shared between fly- and manure-derived isolates and across multiple taxa, providing further evidence for the environmental dissemination of ARG-carrying plasmids.

Collectively, our findings provide compelling evidence for the circulation of both individual bacterial strains and plasmids between *Stomoxys* flies and bovine manure on dairy farms. Although this study likely underrepresents the full extent of strain diversity due to its limited geographic scope, it represents the first comparative genomic analysis of *Stomoxys*-associated bacterial isolates. Our results strongly support the acquisition of fecal bacteria by muscid flies in dairy environments and highlight the high degree of microbiome overlap between flies and manure. These findings underscore the potential role of *Stomoxys* flies as vectors in the environmental dissemination of both commensal and pathogenic bacteria. Further experimental work is needed to determine whether *Stomoxys* flies function solely as passive carriers of manure-associated microbes or if they can actively transmit mastitis-causing bacteria to dairy cattle. Such studies will be critical for evaluating the biosafety risks posed by flies and other arthropods in livestock production systems.

## MATERIALS AND METHODS

### Collection and sequencing of bacterial isolates

Bacterial isolates sequenced in this study were collected as part of three previous studies to characterize the microbiota of biting *Stomoxys* flies in relation to environmental manure piles (7, 8, 34). Detailed methodology related to the collection of *Stomoxys* flies and environmental manure as well as subsequent bacterial isolations is reported in these studies. In brief, sampling was performed on a weekly basis from July 2021 to September 2021 across two farms within the same herd management system in Wisconsin: the Dairy Cattle Center (DCC) and Emmons Blaine Arlington Dairy Research Center (Arlington). Flies were collected on adhesive alsynite fiberglass traps (Olson Products, Medina, OH, USA) set outside the main barn structures at each site. Parallel manure samples were collected from manure scraper systems (Arlington), straw-bedding isolation pens (Arlington), calf pens (Arlington), or as fresh manure (DCC). Retrieved flies were sterilized via repeated washes of ethanol and water and pooled for homogenization, while manure samples were homogenized directly in sterile water. Selective isolation of bacterial taxa was performed on all homogenates, as described previously (7, 8, 34). Previous analysis of these culture results indicates that there was no difference in genus level diversity between flies captured during the early versus late season at either farm (8).

Here, we selected a subset of these isolates for short-read whole genome sequencing, with representative isolates selected from each unique fly pool and manure sample. DNA from the selected isolates was extracted using a NucleoSpin Tissue kit (Macherey-Nagel, Nordrhein-Westfalen, Germany) and quantified using a Quantus fluorometer (Promega, Madison, WI, USA). Genomic DNA sent to SeqCoast Genomics (Portsmouth, NH, USA) was prepared using the Illumina DNA Prep tagmentation kit (San Diego, CA, USA) and sequenced on the NextSeq2000 platform using a 300-cycle flow cell kit to produce 2 × 150 bp paired reads with an estimated 1.3 million reads per sample. Read demultiplexing and read trimming were performed using DRAGEN v3.10.12 as part of the SeqCoast Genomics pipeline. Genomic DNA sent to the UW-Madison Biotechnology Center was prepared using the Celero EZ DNA-Seq prep kit (Tecan Genomics, Männedorf, Switzerland) and sequenced on the Illumina NovaSeq6000 platform to generate 2 × 150 bp shared reads with an estimated 1 million reads per sample. Removal of adapter and low-quality sequences was performed using Trimmomatic (v0.39) (82). Sequence quality was checked on both raw and trimmed reads using FastQC (v0.11.9) (83).

### Genome assembly, annotation, and quality control

Genome assembly was performed using the SPAdes assembler (v3.15.5) (84) as implemented through Shovill (v1.1.0) (85). Assembly statistics were determined using QUAST (v5.2.0), and detection of expected universal single copy orthologs was performed using BUSCO (v 5.7.1) (35, 36). The completeness and contamination levels of draft assemblies were assessed using the CheckM (v1.0.18) full reference tree lineage workflow on the KBase platform (86, 87). All assembly statistics are available as supplemental materials (Table S1). Contigs were annotated using the Prokka pipeline (v 1.13) implemented with the optional “rfam” computation setting (88).

### Taxonomic classification and typing of isolates

Bacterial isolates were identified by comparing draft assemblies to the PubMLST ribosomal multilocus sequence typing (rMLST) database, which resolves taxonomy based on 53 ribosome protein subunit (*rps*) genes (89). For identified *E. coli* isolates, sequence types were determined through comparison of allelic profiles against the Pasteur eight gene MLST scheme, and the phylogroup was predicted using the ClermonTyping program (42, 90). Furthermore, *in silico E. coli* serotyping was performed via the Center for Genomic Epidemiology (CFGE) SerotypeFinder (v 2.0.1) (91). For isolates identified as *K. pneumoniae*, Kleborate (v2.4.1; Kaptive setting) was used to determine the sequence type and to predict the K (capsule) and O antigen (LPS) serotype (45, 92). ABRicate (v1.01; databases downloaded 2024-Jun-11) was used to screen all contigs for the presence of plasmid replicon genes (*rep*) (PlasmidFinder), VFs (VFDB; VirulenceFinder for *E. coli* isolates), and ARGs (Resfinder) (37–41).

### Phylogenetic analysis in a local and global context

Phylogenetic analysis was performed for the most highly represented taxonomic groups in the collection (*E. coli*, *K. pneumoniae*, *M. sciuri*, and *Staphylococcus* spp.). Panaroo (v1.5.0) was used to produce core genome alignment (core_threshold 0.98; MAFFT) files for each listed taxonomic group (93, 94). Maximum-likelihood phylogenetic trees were inferred on filtered core genome alignments using a Jukes-Cantor+CAT nucleotide substitution model via FastTree (v2.1.11) (95). Reference genome sets were constructed for each taxonomic group to determine the global phylogenetic placement of sequenced isolates (Table S2). For *E. coli*, a reference genome set was constructed based on available genomes from the *Escherichia coli* reference collection (ECOR) (96, 97), and further supplemented with select pathotype genomes as well as mastitis- and fly-derived isolates. For *K. pneumoniae*, genomes were compiled from a previously described reference set along with available mastitis- and fly-derived genomes (74). For both *M. sciuri* and *Staphylococcus* spp., the initial reference sets included all respective publicly available genomes from the NCBI Genome database. The drep genome comparison tool (dRep compare --P_ani0.999 S_ani .999; v3.5.0) was used to identify highly similar genomes within each reference set based on both MASH and ANI (98). To minimize duplication of strains, a representative genome was selected when two or more genomes from the same environment and submission details (BioProject) shared more than 99.99% average nucleotide identity as calculated through fastANI (v 1.33) (99). Global phylogenies were constructed based on annotated genomes using Panaroo and FastTree, as described above (88, 93, 95). Bacterial phylogenies were visualized in the “ggtree” package and the TreeViewer software.

### Identification of clustering based on pangenome features and functional annotations

Bacterial pangenome features were imported into R for further analysis using the “Pagoo” package (93, 100). A principal component analysis was performed on the pangenome matrix using the ‘pagoo::pan_pca()’ function, and associated clustering analysis was performed using the k-means algorithm via the “factoextra” package (v1.0.7) (101). To test for host association with specific clusters, we performed Chi-square tests (simulated *P*-values with 10,000 Monte Carlo simulations) and visualized the standardized residuals via the “pheatmap” (v1.0.12) package in R (102).

Further functional annotation was performed to identify orthologs associated with identified bacterial clusters or lineages. Predicted protein sequences were used as input into eggNOG-mapper (v2.1.12) to perform a diamond BLAST (v2.1.10) sequence alignment against the eggnog protein database (v5.0.2) with a minimum hit *e*-value of 0.001 (47–49). Resulting annotation files were imported into R (https://www.r-project.org) to compute counts of identified KEGG Enzyme (EC) numbers (103, 104). Chi-square tests (simulated *P*-values with 10,000 Monte Carlo simulations) were performed on calculated contingency tables to test for significant associations between functional counts and taxonomic clusters. Descriptive information for significant ECs was attached using the “KEGGREST” package and calculated standardized residuals were visualized using “ggplot2” (105, 106).

### Identification of highly similar *Staphylococcaceae* genomes and plasmids

We further screened phylogenetic trees to identify clustered pairs of phylogenetically related sequenced isolates from different sources (fly, manure, and mastitis). Pairwise genome comparisons were performed on select genome pairs using MUMmer4 (nucmer v4.0.0, DNAdiff v1.3) (50). To determine the potential transmission of small ARG-encoding plasmids throughout the environment, contig sequences containing both a plasmid replicon gene and an associated ARG were extracted from *Staphylococcaceae* draft genome assemblies. For comparison, contigs were re-oriented to include all *rep* genes in the forward direction. Plasmids were sorted by *rep* type (as identified above by PlasmidFinder), and subsequent pairwise sequence alignments to determine percent similarity were performed using MUMmer4 (v4.0.0) as implemented in MobMess (39, 50, 51). The resulting pairwise global percent similarities were visualized in R using the “ComplexHeatmap” package (107). Plasmid maps were constructed using “gggenomes” on reindexed annotated (Bakta v1.8.2) contig sequences to include *rep* genes in the starting position for easier visualization (108, 109).

## Data Availability

Raw Illumina reads and assemblies are available in the NCBI Sequence Read Archive (https://www.ncbi.nlm.nih.gov/sra) under BioProject ID PRJNA1216036. Data supporting the conclusions of this article, along with scripts used for analysis and figure generation, are provided as supplemental materials or are available in the Coon laboratory’s GitHub repository (https://github.com/kcoonlab/stable-fly-genomes). Physical isolates are available upon request.

## SUPPLEMENTAL MATERIAL

Supplemental figures msystems.00215-25-s0001.pdf Figures S1 to S12.

Table S1 msystems.00215-25-s0002.xlsx Genome assembly and quality statistics.

Table S2 msystems.00215-25-s0003.xlsx Screening of all contigs for VFs, ARGs, and plasmid *rep* genes.

Table S3 msystems.00215-25-s0004.xlsx Further typing of *E. coli* isolates and associated metadata related to Fig. 3.

Table S4 msystems.00215-25-s0005.xlsx Construction of reference sets for phylogenetic placement of isolates.

Table S5 msystems.00215-25-s0006.xlsx Further typing of *K. pneumoniae* isolates and associated metadata related to Fig. 5.

Table S6 msystems.00215-25-s0007.xlsx Chi-square residuals and prevalence data for *M. sciuri* KEGG ECs.

Table S7 msystems.00215-25-s0008.xlsx MUMmer4 pairwise genome analysis and analysis of unaligned contigs.

Table S8 msystems.00215-25-s0009.xlsx Co-occurrence of ARGs and plasmid *rep* genes in *Staphylococcaceae* isolates.

ASM does not own the copyrights to Supplemental Material that may be linked to, or accessed through, an article. The authors have granted ASM a non-exclusive, world-wide license to publish the Supplemental Material files. Please contact the corresponding author directly for reuse.
