# Supplementary material for: Phylogenetic intermixing reveals stable fly-mediated circulation of mastitis-associated bacteria in dairy settings
Source: mSystems. 2025 Aug 1;10(9):e00215-25. doi: 10.1128/msystems.00215-25 (PMC12455971; doi:10.1128/msystems.00215-25)
Supplement: Supplemental figures — Figures S1 to S12. [file msystems.00215-25-s0001.pdf]

**Supplemental figures for**: Phylogenetic intermixing reveals stable fly-mediated circulation of mastitis-associated bacteria in dairy settings

Andrew J. Sommer[1,2#], Travis K. Worley[1,2], Panagiotis Sapountzis[3], Kerri L. Coon[1#]

[1]Department of Bacteriology, University of Wisconsin-Madison, Madison, WI 53706
[2]Microbiology Doctoral Training Program, University of Wisconsin-Madison, Madison, WI 53706
[3]INRAE-UCA, UMR0454 MEDIS, Clermont-Ferrand, France

[#]Correspondence: Andrew J. Sommer, asommer101@gmail.com
Kerri L. Coon, kerri.coon@wisc.edu

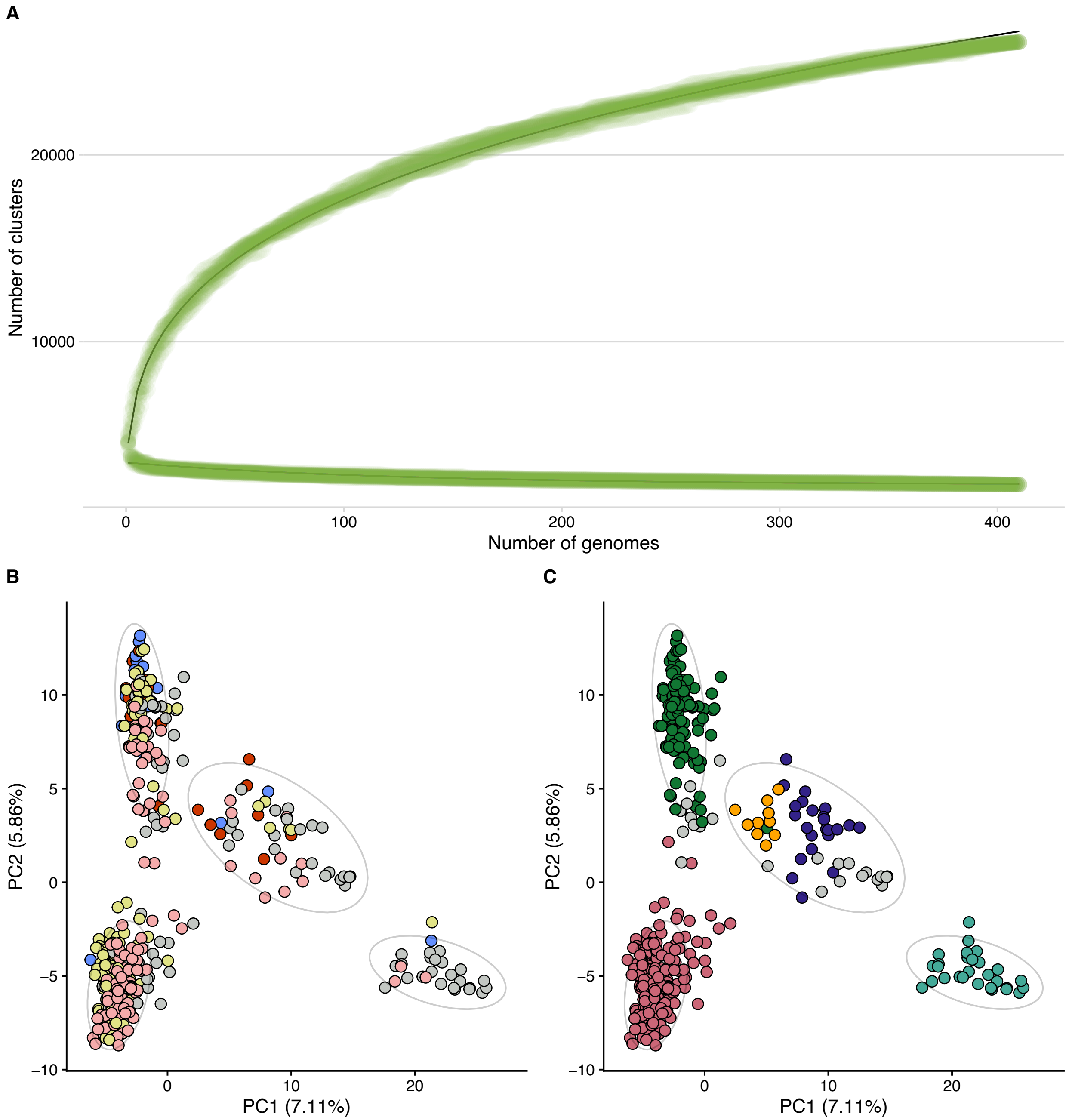


**Figure S1.** *E. coli* pangenome analysis. (A) Pan and core genome curves as calculated by Pagoo. (B/C) PCA visualizations based on gene content. Figure S1B is colored by sample origin as follows: flies (red), manure (blue), mastitis (yellow), other (grey). The dark red dots indicate isolates sequenced in this study; the light red dots correspond to publicly available fly-derived *E. coli* genomes. Figure S1C is colored by MASH phylogroup as follows: D (Dark blue), B2 (cyan), E (orange), A (light red), B1 (green), other (grey).

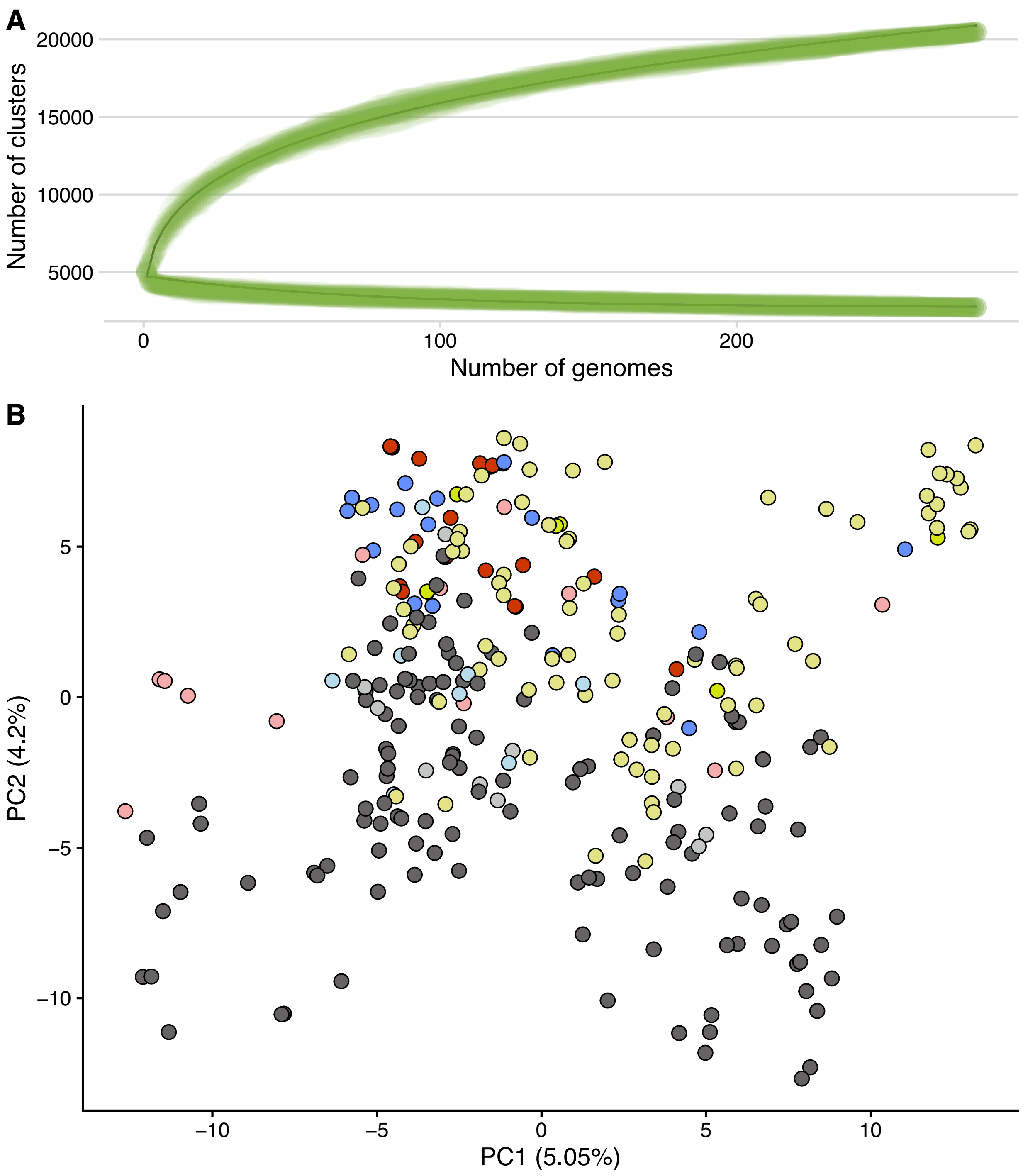


**Figure S2.** *K. pneumoniae* pangenome analysis. (A) Pan and core genome curves as calculated by Pagoo. (B) PCA visualizations based on gene content. The color corresponds to the sample origin as follows: flies (red), manure (blue), mastitis (yellow), human (dark grey), other (grey). The dark red dots indicate isolates sequenced in this study; the light red dots correspond to publicly available fly-derived genomes.

A.

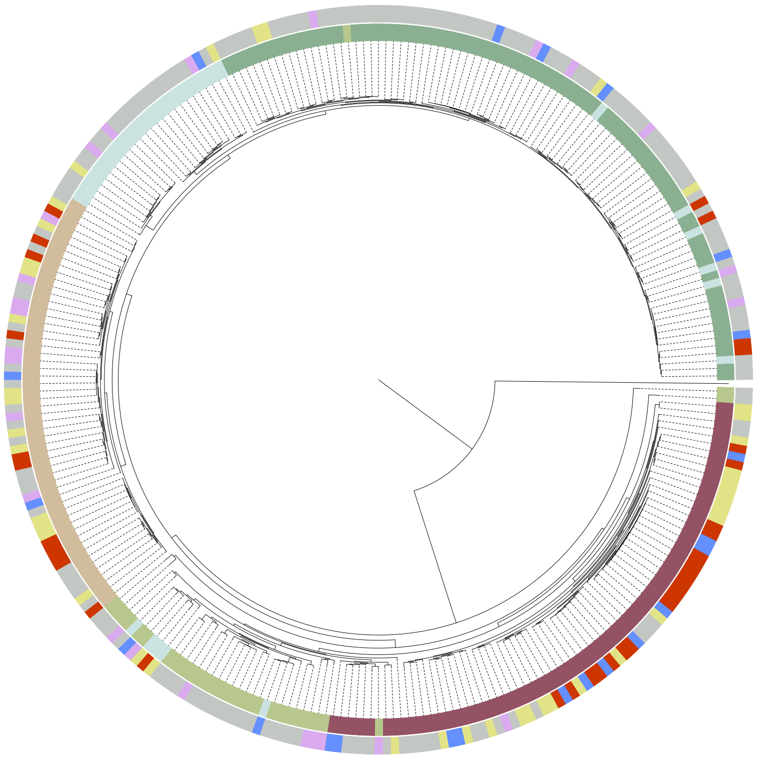

B.

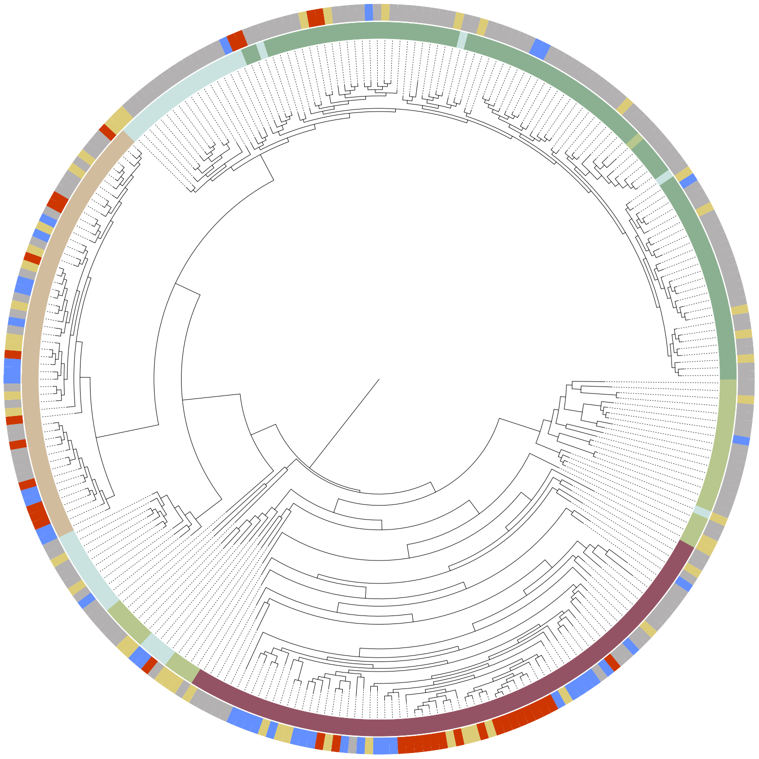

[Figure caption on next page]

**Figure S3.** *M. sciuri* supporting phylogenetic trees. (A) Outgroup rooted maximum-likelihood phylogenetic tree based on alignment of six housekeeping genes. Tree is rooted with an *S. xylosus* isolate as an outgroup. (B) Midpoint rooted maximum-likelihood phylogenetic tree based on alignment of core genes. For both trees, the exterior rectangle node annotation corresponds to the sample origin and the interior node annotation corresponds to the cluster as assigned by gene content (See Figure 6 legend).

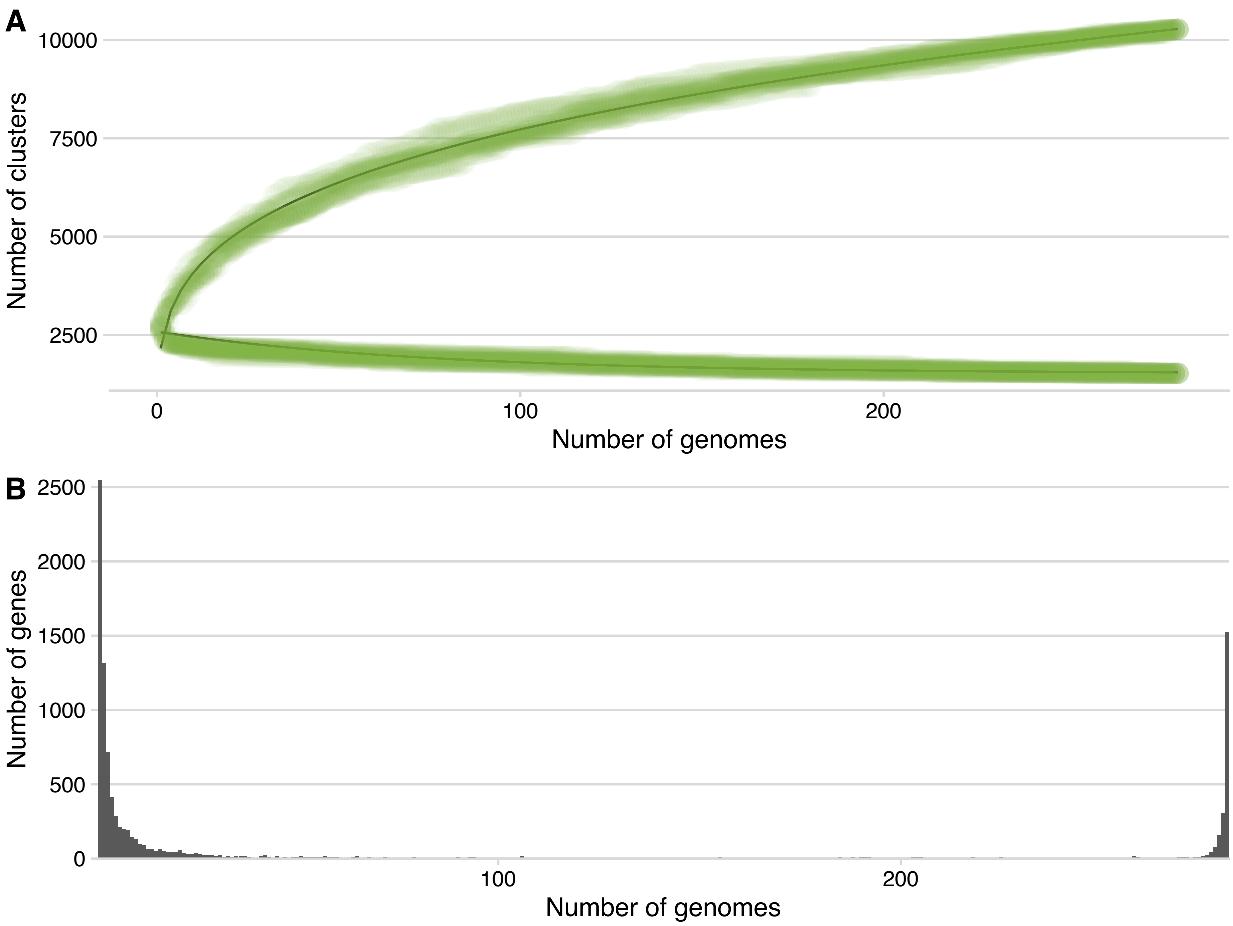


**Figure S4.** *M. sciuri* pangenome analysis supporting figures. (A) Pan and core genome curves as calculated by Pagoo. (B) Barplot showing the frequency distribution of identified gene clusters in the *M. sciuri* pangenome.

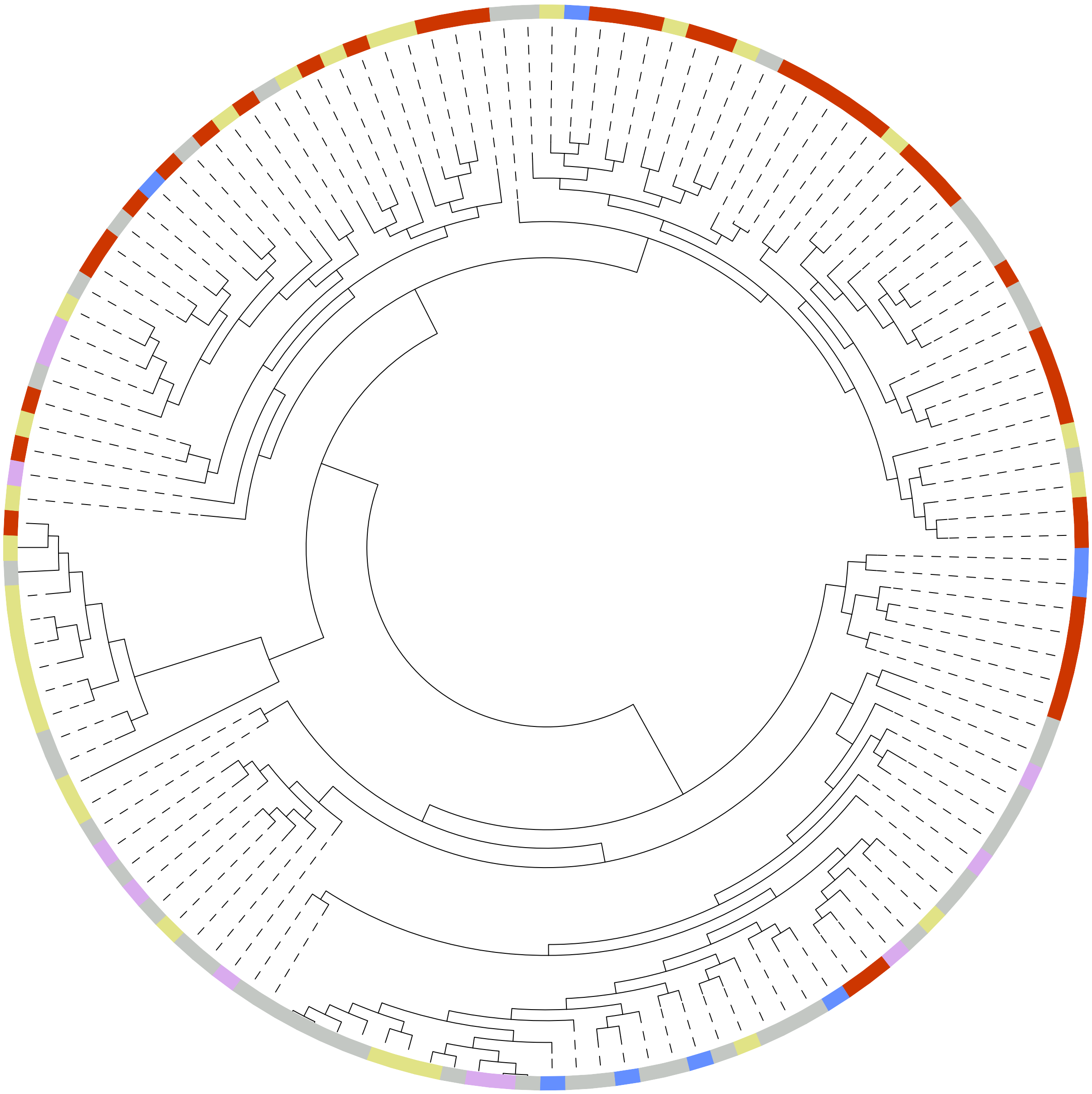

**Figure S5:** Phylogenetic tree of sequenced *S. xylosus* isolates and NCBI reference strains. Phylogeny is shown as midpoint rooted maximum-likelihood trees based on a core genome alignment. Sample origins are colored as follows: flies (red), manure or cattle gastrointestinal tract (blue), bovine mastitis (yellow), cattle skin or nasal (purple), other (grey).

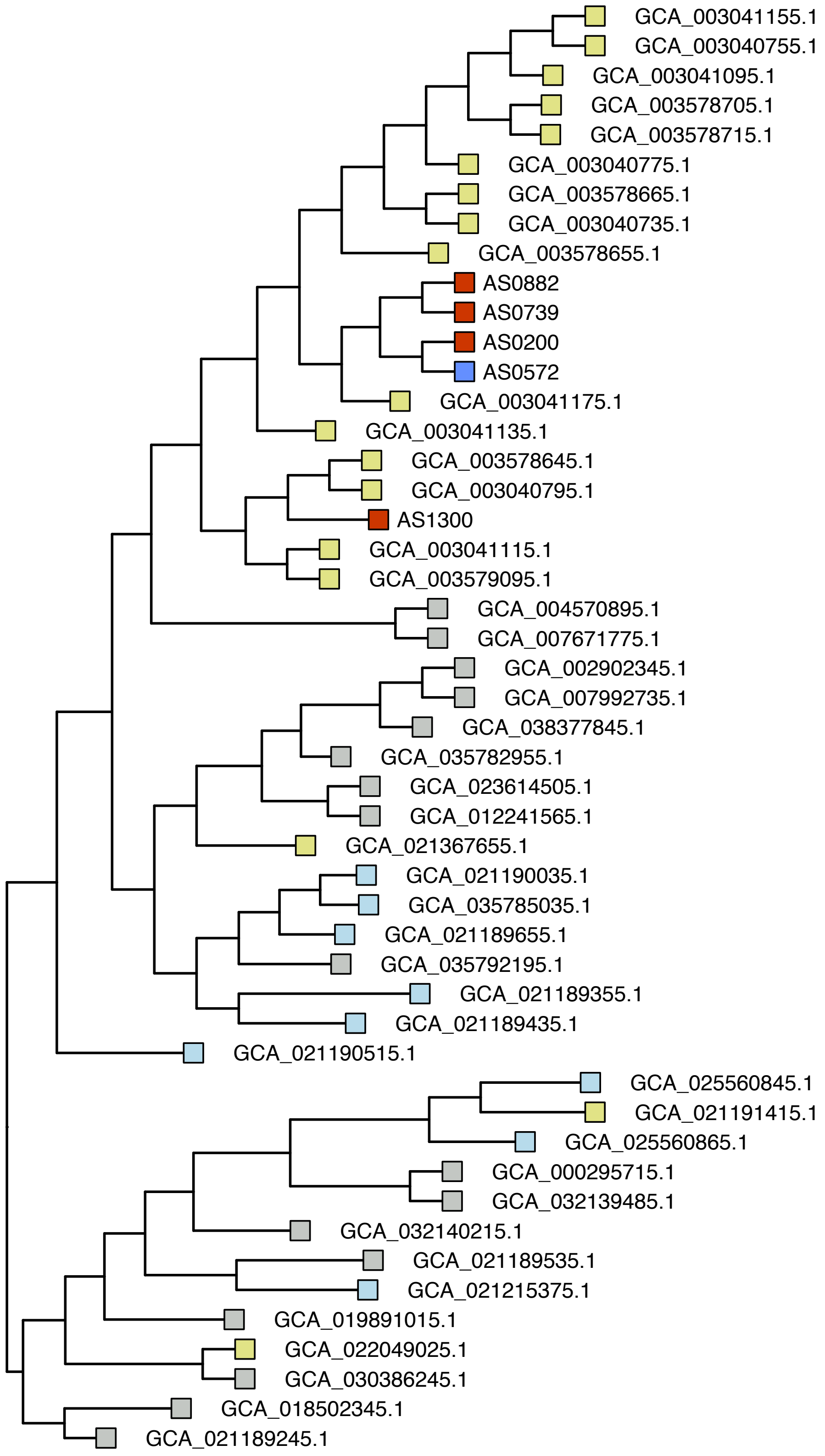


[Figure caption on next page]

**Figure S6:** Phylogenetic tree of sequenced *S. arlettae* isolates and NCBI reference strains. Phylogeny is shown as midpoint rooted maximum-likelihood trees based on a core genome alignment. Sample origins are colored as follows: flies (red), manure or cattle gastrointestinal tract (blue), bovine mastitis (yellow), other (grey). Manure strains sequenced in this study are represented by the darker blue node; publicly available manure strains are represented by the lighter blue node.

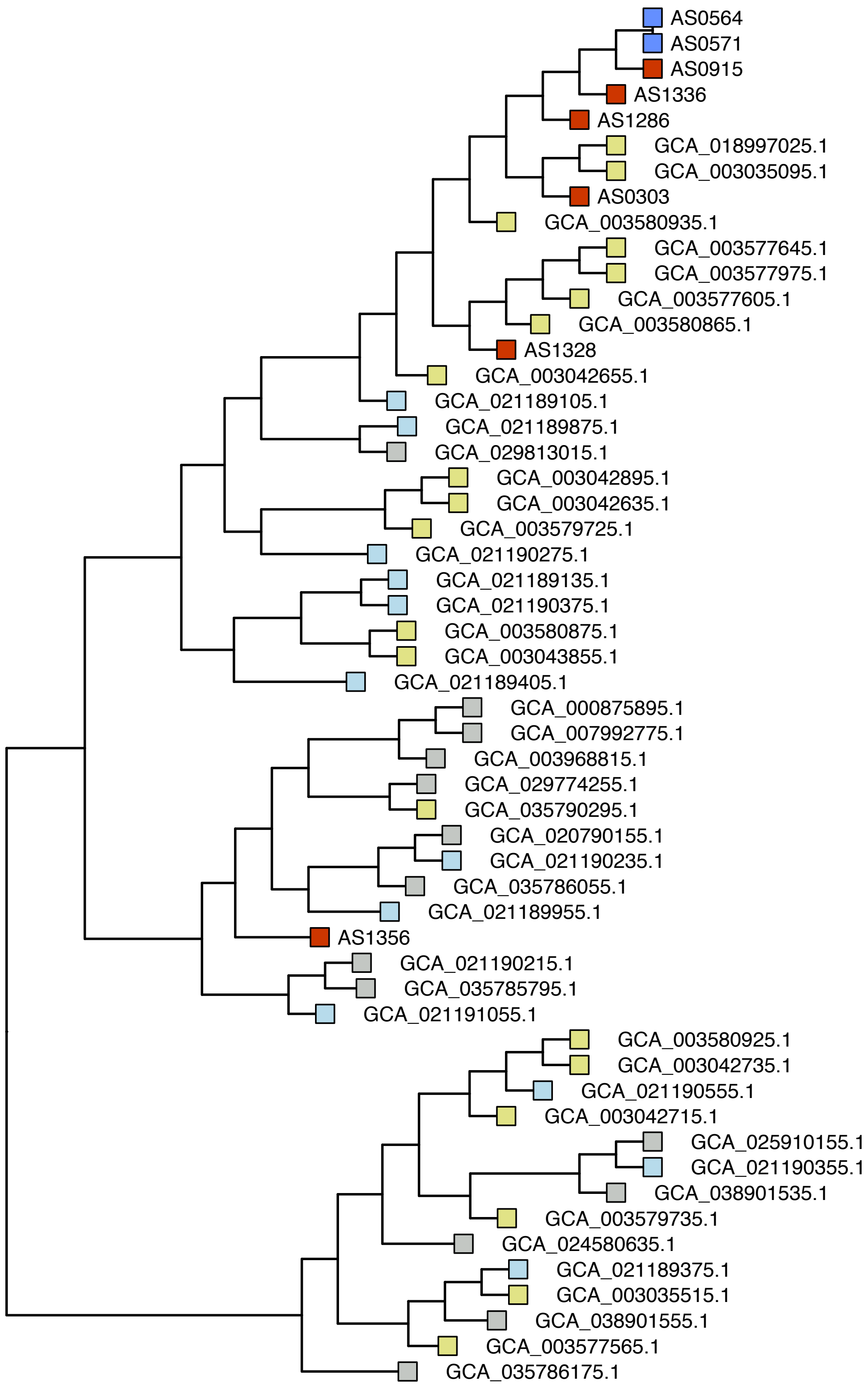


[Figure caption on next page]

**Figure S7:** Phylogenetic tree of sequenced *S. gallinarum* isolates and NCBI reference strains. Phylogeny is shown as midpoint rooted maximum-likelihood trees based on a core genome alignment. Sample origins are colored as follows: flies (red), manure or cattle gastrointestinal tract (blue), bovine mastitis (yellow), other (grey). Manure strains sequenced in this study are represented by the darker blue node; publicly available manure strains are represented by the lighter blue node.

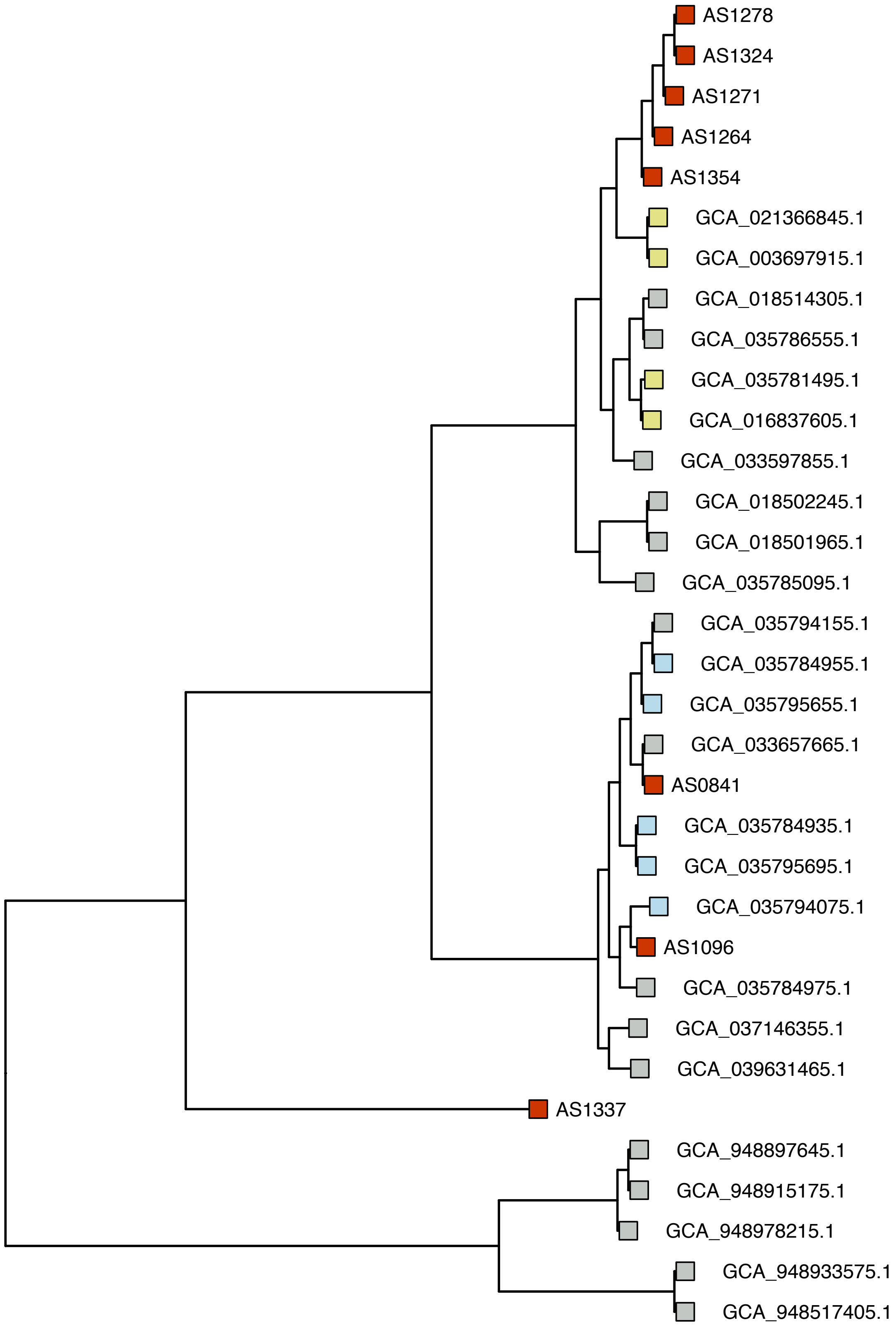


[Figure caption on next page]

**Figure S8:** Phylogenetic tree of sequenced *S. pseudoxylosus* isolates and NCBI reference strains. Phylogeny is shown as midpoint rooted maximum-likelihood trees based on a core genome alignment. Sample origins are colored as follows: flies (red), manure or cattle gastrointestinal tract (blue), bovine mastitis (yellow), other (grey). No manure *S. pseudoxylosus* strains were sequenced in this study.

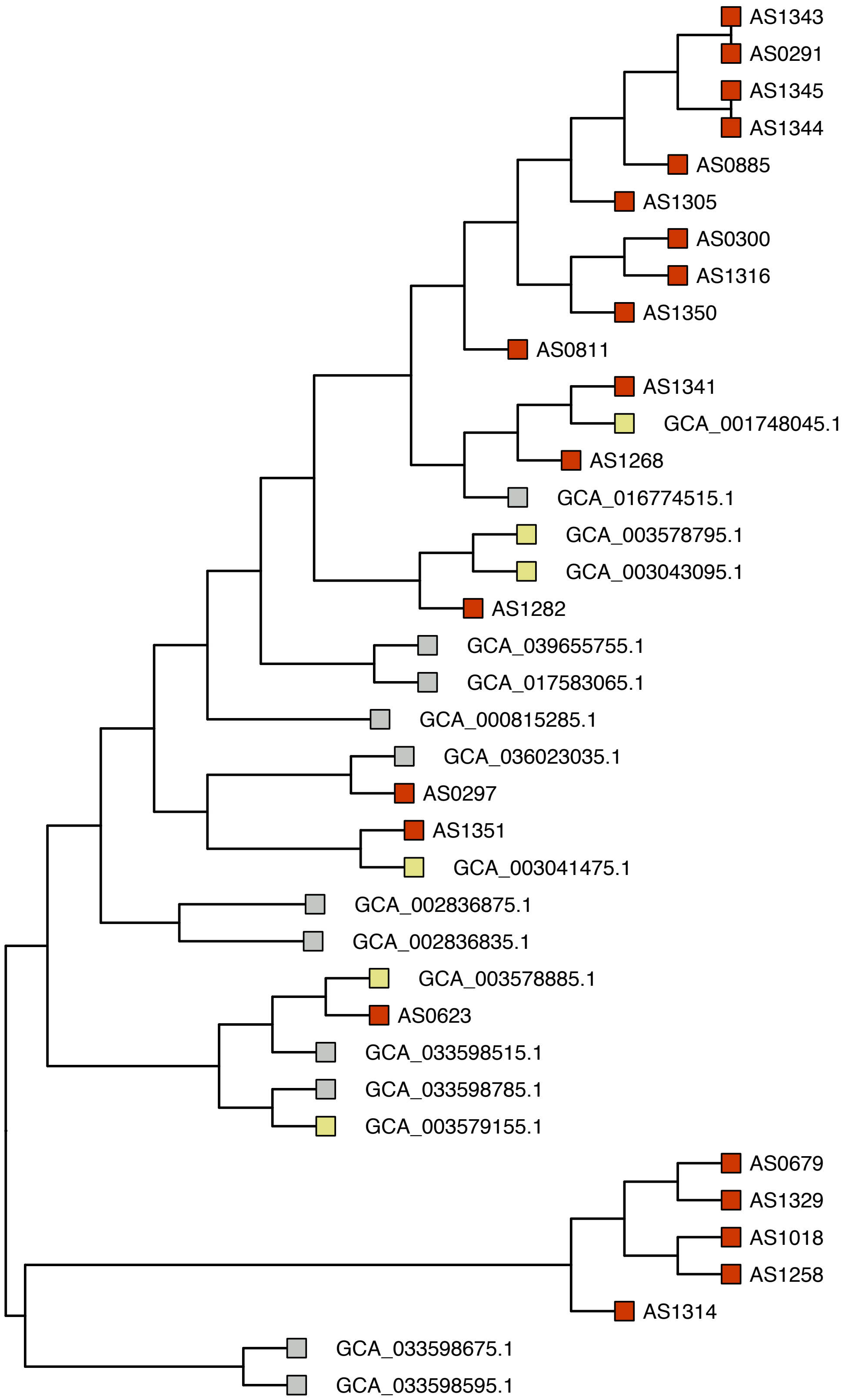


[Figure caption on next page]

**Figure S9:** Phylogenetic tree of sequenced *S. shinii* isolates and NCBI reference strains. Phylogeny is shown as midpoint rooted maximum-likelihood trees based on a core genome alignment. Sample origins are colored as follows: flies (red), bovine mastitis (yellow), other (grey).

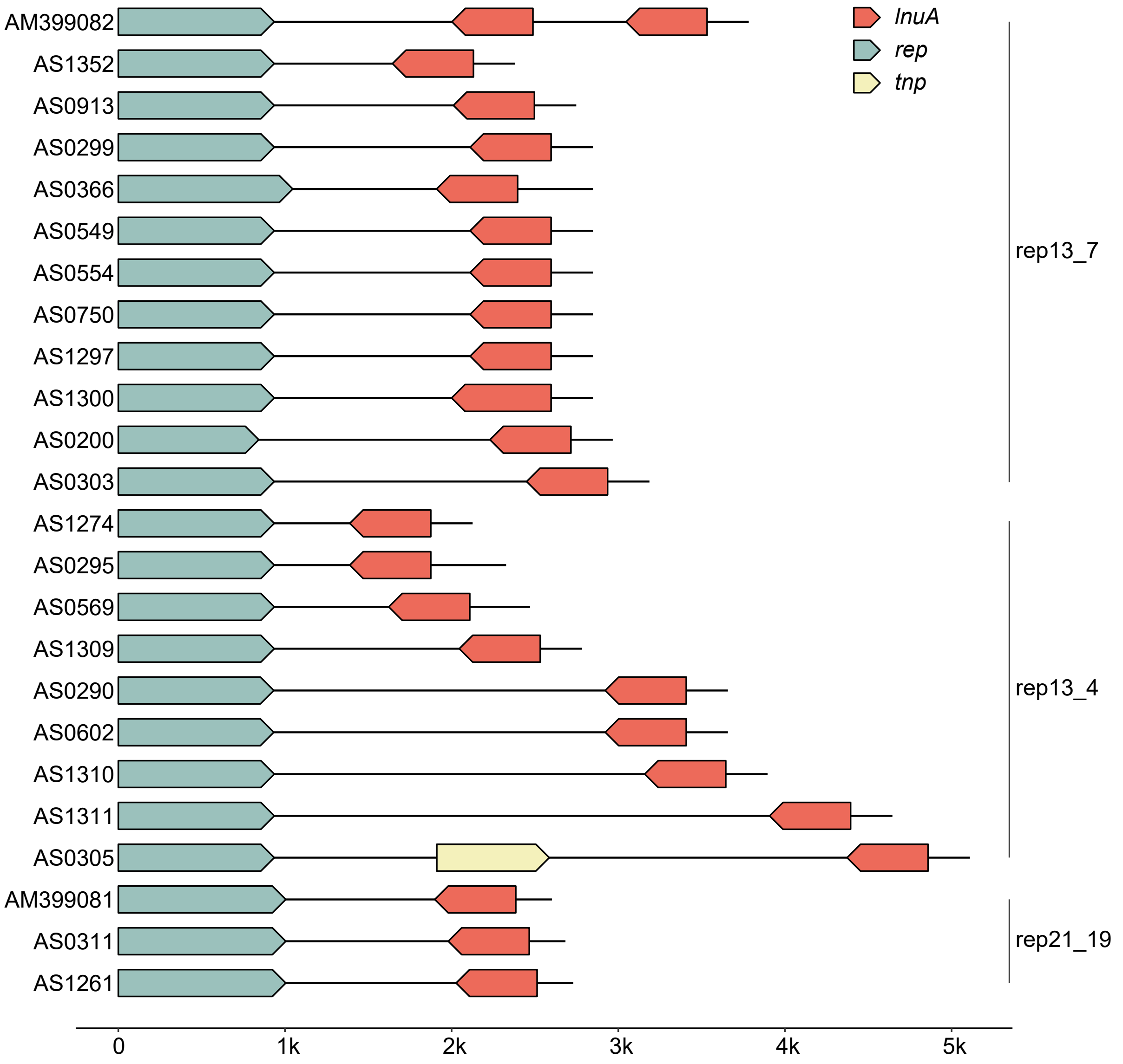


**Figure S10.** The genomic architecture of *lnuA* encoding plasmids. The figure depicts schematic diagrams indicating the location of *lnuA*, *rep* (replicon protein gene), and *tnp* genes on plasmids identified from sequenced *Staphylococcaceae* strains along with selected reference sequences. All sequences were reindexed to position the rep gene on the left-hand side of the forward strand. The rep gene variant (identified via PlasmidFinder) is noted.

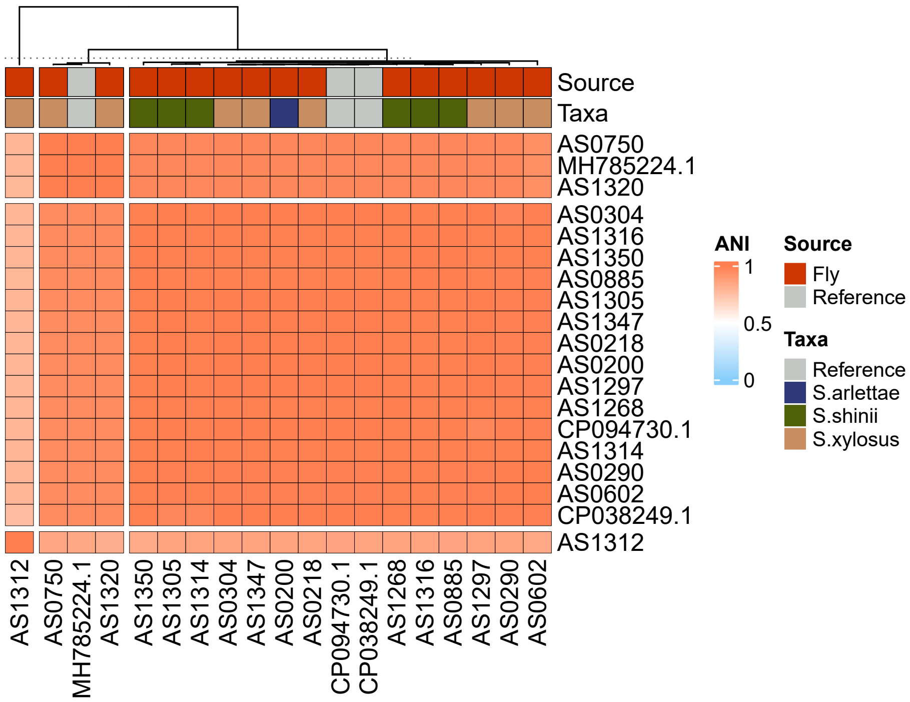


**Figure S11.** Heatmap depicting the global percent nucleotide similarity of plasmid-associated contigs encoding the *tetK* gene. Percent similarity and associated strain metadata (taxonomic identification, strain origin) are marked as described in Figure 7. MH785224.1, CP094730.1, and CP038249.1 are included as reference strains.

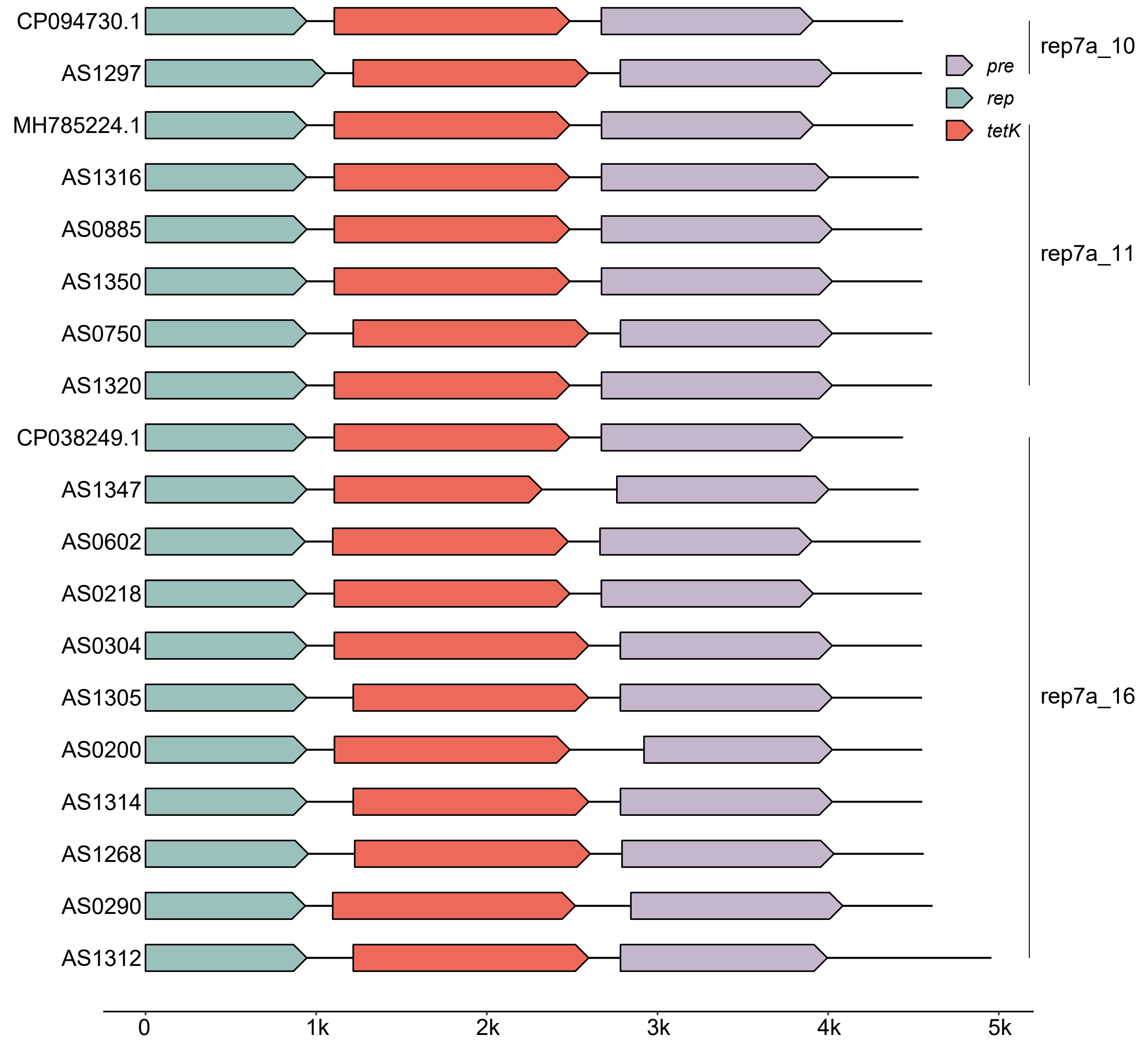


**Figure S12.** The genomic architecture of *tetK* encoding plasmids. The figure depicts schematic diagrams indicating the location of *tetK*, *rep* (replicon protein gene), and *pre* (plasmid recombination enzyme) genes on plasmids identified from sequenced Staphylococcaceae strains along with selected reference sequences. All sequences were reindexed to position the rep gene on the left-hand side of the forward strand. The *rep* gene variant (identified via PlasmidFinder) is noted on the right-hand side.
